# Supplementary material for: Healthy food retail availability and cardiovascular mortality in the United States: a cohort study
Source: BMJ Open. 2021 Jul 9;11(7):e048390. doi: 10.1136/bmjopen-2020-048390 (PMC8273445; doi:10.1136/bmjopen-2020-048390)
Supplement: Supplementary data [file bmjopen-2020-048390supp001.pdf]

BMJ Publishing Group Limited (BMJ) disclaims all liability and responsibility arising from any reliance placed on this supplemental material which has been supplied by the author(s)

Healthy Food Retail Availability and Cardiovascular Mortality Using Linked Data across the Contiguous US from the Mortality Disparities in American Communities Study (Supplemental materials)

INTRODUCTION TO SUPPLEMENTAL TABLES AND FIGURE

Sex stratified analyses (Tables S1 and S2) and analyses restricted to urban residents (Table S3) and households with no more than one subfamily (Table S4) follow the format of Table 3, and Figure S1 depicts at a glance how these compare to the main analysis finding.

Also following a format parallel to Table 3, the following tables show results from frailty analyses to account for clustering by county (S5) and using census tract data instead of ZCTA data (S6).

Lovasi GS, *et al. BMJ Open* 2021; 11:e048390. doi: 10.1136/bmjopen-2020-048390

Figure S1. Hazard ratios and 95% confidence intervals for association between healthy food availability at the ZCTA level and cardiovascular mortality, across adjustment and subgroups

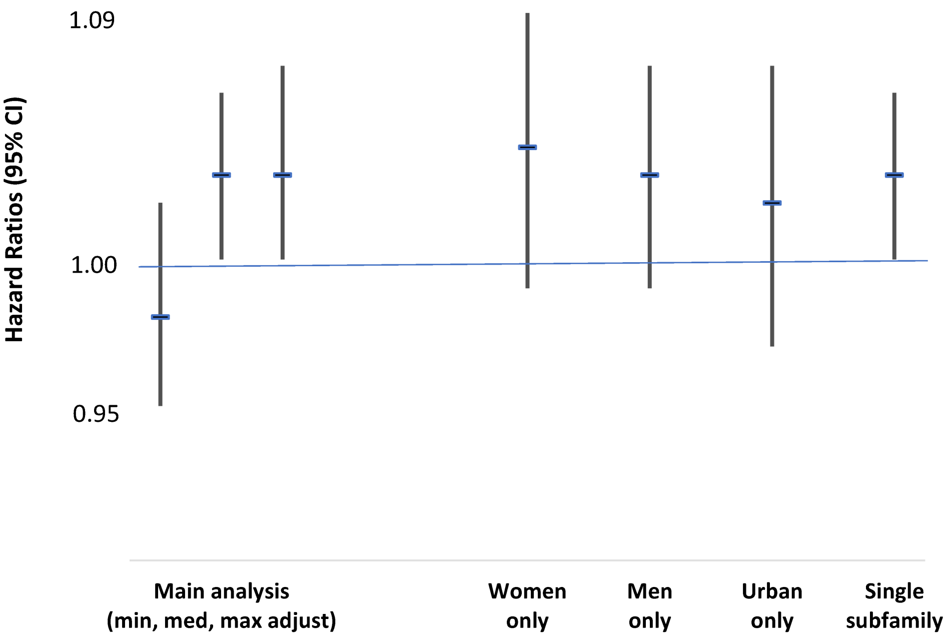

Notes: Values show are hazard ratios and 95% confidence intervals from models of healthy food retail presence with cardiovascular mortality, where “min” indicates minimally adjusted main analysis models which included gender, age, marital status, nativity, Black race, and Hispanic ethnicity; “med” indicates moderately adjusted main analysis models which included adjustment all covariates in “min” plus educational attainment and income; and “max” indicates maximally adjusted models adjusted for gender (except in gender stratified models), age, marital status, nativity, Black race, Hispanic ethnicity, educational attainment, income, median household income, population density, and walkable destination density; N=2,753,000 for main analysis, and the N is reduced for maximally adjusted stratum-specific models (1,461,000 among women, 1,292,000 among men, 1,911,000 among urban residents, and 2,711,000 among single subfamily households); CBDRB-FY20-CES004-030

Lovasi GS, *et al. BMJ Open* 2021; 11:e048390. doi: 10.1136/bmjopen-2020-048390

Table S1. Hazard ratios and 95% confidence intervals for association with cardiovascular mortality among women

	Minimally adjusted	Moderate adjustment	Fully Adjusted
Any supermarket or produce market present	1.01 (0.99-1.06)	1.04 (0.99-1.09)	1.04 (0.99-1.09)
Age (rescaled to per 10 years)	**2.93 (2.89-2.97)**	**2.84 (2.81-2.88)**	**2.84 (2.81-2.88)**
Married	**0.62 (0.60-0.65)**	**0.66 (0.64-0.69)**	**0.67 (0.64-0.69)**
US born	**1.24 (1.18-1.31)**	**1.20 (1.13-1.27)**	**1.22 (1.15-1.29)**
Black race	**1.12 (1.06-1.17)**	**1.07 (1.01-1.12)**	0.98 (0.93-1.04)
Hispanic ethnicity	**0.91 (0.84-0.97)**	**0.85 (0.79-0.91)**	**0.79 (0.74-0.85)**
Educational attainment college or more		**0.62 (0.59-0.66)**	**0.63 (0.60-0.67)**
Income (rescaled to per 10K)		**0.98 (0.97-0.98)**	**0.98 (0.98-0.98)**
Median household income (rescaled to per 10K)			**0.96 (0.95-0.97)**
Population density (residents/km^2) (rescaled to per 10K/km^2)	(shaded indicates exclude from model)		**1.20 (1.12-1.29)**
Walkable destination density (count/km^2), (rescaled to per SD)			0.99 (0.96-1.01)

Notes: Values show in each cell are hazard ratios and 95% confidence intervals from models with N=1,461,000 women; Boldface indicates statistical significance ($p<0.05$); CBDRB-FY20-CES004-030

Table S2. Hazard ratios and 95% confidence intervals for association with cardiovascular mortality among men

	Minimally adjusted	Moderate adjustment	Fully Adjusted
Any supermarket or produce market present	0.97 (0.93-1.01)	1.03 (0.98-1.07)	1.03 (0.99-1.07)
Age (rescaled to per 10 years)	**2.59 (2.57-2.62)**	**2.52 (2.49-2.54)**	**2.52 (2.49-2.55)**
Married	**0.58 (0.56-0.60)**	**0.63 (0.61-0.65)**	**0.63 (0.61-0.65)**
US born	**1.14 (1.37-1.52)**	**1.40 (1.33-1.47)**	**1.39 (1.32-1.47)**
Black race	**1.07 (1.02-1.12)**	0.96 (0.92-1.01)	**0.92 (0.87-0.96)**
Hispanic ethnicity	**0.87 (0.82-0.93)**	**0.77 (0.73-0.82)**	**0.75 (0.70-0.79)**
Educational attainment college or more		**0.67 (0.65-0.70)**	**0.69 (0.66-0.72)**
Income (rescaled to per 10K)		**0.97 (0.97-0.97)**	**0.97 (0.97-0.98)**
Median household income (rescaled to per 10K)			**0.96 (0.96-0.97)**
Population density (residents/km^2) (rescaled to per 10K/km^2)	(shaded indicates exclude from model)		1.04 (0.97-1.11)
Walkable destination density (count/km^2), (rescaled to per SD)			1.01 (0.99-1.03)

Notes: Values show in each cell are hazard ratios and 95% confidence intervals from models with N=1,292,000 men; Boldface indicates statistical significance ($p<0.05$); CBDRB-FY20-CES004-030

Table S3. Hazard ratios and 95% confidence intervals for association with cardiovascular mortality among urban residents

	Minimally adjusted	Moderate adjustment	Fully Adjusted
Any supermarket or produce market present	1.02 (0.97-1.08)	1.03 (0.98-1.09)	1.02 (0.97-1.07)
Female gender	**0.45 (0.44-0.46)**	**0.43 (0.42-0.44)**	**0.43 (0.42-0.45)**
Age (rescaled to per 10 years)	**2.73 (2.70-2.75)**	**2.65 (2.63-2.67)**	**2.65 (2.63-2.67)**
Married	**0.58 (0.57-0.60)**	**0.64 (0.62-0.65)**	**0.64 (0.62-0.66)**
US born	**1.35 (1.30-1.40)**	**1.32 (1.27-1.37)**	**1.33 (1.28-1.38)**
Black race	**1.11 (1.07-1.16)**	1.03 (0.99-1.07)	**0.96 (0.92-1.00)**
Hispanic ethnicity	**0.90 (0.85-0.94)**	**0.81 (0.77-0.85)**	**0.77 (0.73-0.81)**
Educational attainment college or more		**0.67 (0.64-0.69)**	**0.68 (0.66-0.70)**
Income (rescaled, e.g., to per 10K or per SD)		**0.97 (0.97-0.98)**	**0.98 (0.98-0.98)**
Median household income (rescaled to per 10K)			**0.96 (0.96-0.97)**
Population density (residents/km^2) (rescaled to per 10K/km^2)	(shaded indicates exclude from model)		**1.11 (1.06-1.17)**
Walkable destination density (count/km^2), (rescaled to per SD)			1.00 (0.98-1.01)

Notes: Values show in each cell are hazard ratios and 95% confidence intervals from models with N=1,911,000 urban residents; Urban was defined by the Census Bureau, based on whether the geography was within an urbanized area or urban cluster; Boldface indicates statistical significance (p<0.05); CBDRB-FY20-CES004-030

Table S4. Hazard ratios and 95% confidence intervals for association with cardiovascular mortality among single family households

	Minimally adjusted	Moderate adjustment	Fully Adjusted
Any supermarket or produce market present	0.98 (0.95-1.01)	1.03 (1.00-1.06)	1.03 (1.00-1.06)
Female gender	**0.44 (0.43-0.46)**	**0.43 (0.42-0.44)**	**0.43 (0.42-0.44)**
Age (rescaled to per 10 years)	**2.72 (2.69-2.74)**	**2.63 (2.61-2.66)**	**2.64 (2.61-2.66)**
Married	**0.57 (0.56-0.59)**	**0.62 (0.61-0.64)**	**0.63 (0.61-0.64)**
US born	**1.34 (1.29-1.39)**	**1.30 (1.25-1.35)**	**1.30 (1.25-1.36)**
Black race	**1.09 (1.05-1.13)**	1.00 (0.97-1.04)	**0.94 (0.91-0.98)**
Hispanic ethnicity	**0.89 (0.85-0.93)**	**0.81 (0.77-0.85)**	**0.77 (0.73-0.81)**
Educational attainment college or more		**0.65 (0.63-0.67)**	**0.67 (0.65-0.69)**
Income (rescaled, e.g., to per 10K or per SD)		**0.97 (0.97-0.98)**	**0.98 (0.97-0.98)**
Median household income (rescaled to per 10K)			**0.96 (0.96-0.97)**
Population density (residents/km^2) (rescaled to per 10K/km^2)	(shaded indicates exclude from model)		**1.12 (1.07-1.17)**
Walkable destination density (count/km^2), (rescaled to per SD)			1.00 (0.98-1.01)

Notes: Values show in each cell are hazard ratios and 95% confidence intervals from models with N=2,711,000 in households with no more than one subfamily; Boldface indicates statistical significance ($p<0.05$); CBDRB-FY20-CES004-030

Lovasi GS, *et al. BMJ Open* 2021; 11:e048390. doi: 10.1136/bmjopen-2020-048390

Table S5. Hazard ratios and 95% confidence intervals for association with cardiovascular mortality, conditional estimates from frailty models accounting for clustering by county

	Minimally adjusted	Moderate adjustment	Fully Adjusted
Any supermarket or produce market present	0.99 (0.96-1.03)	1.02 (0.98-1.05)	1.02 (0.99-1.05)
Female gender	**0.44 (0.43-0.45)**	**0.43 (0.42-0.44)**	**0.43 (0.42-0.44)**
Age (rescaled to per 10 years)	**2.71 (2.69-2.73)**	**2.63 (2.61-2.65)**	**2.63 (2.61-2.66)**
Married	**0.58 (0.57-0.59)**	**0.63 (0.61-0.64)**	**0.63 (0.62-0.65)**
US born	**1.38 (1.33-1.44)**	**1.38 (1.33-1.44)**	**1.38 (1.32-1.43)**
Black race	**1.05 (1.01-1.09)**	0.97 (0.93-1.01)	**0.92 (0.89-0.96)**
Hispanic ethnicity	**0.83 (0.79-0.87)**	**0.75 (0.71-0.78)**	**0.72 (0.69-0.76)**
Educational attainment college or more		**0.66 (0.64-0.68)**	**0.67 (0.65-0.69)**
Income (rescaled, e.g., to per 10K or per SD)		**0.97 (0.97-0.98)**	**0.98 (0.97-0.98)**
Median household income (rescaled to per 10K)			**0.96 (0.95-0.96)**
Population density (residents/km^2) (rescaled to per 10K/km^2)	(shaded indicates exclude from model)		0.95 (0.89-1.11)
Walkable destination density (count/km^2), (rescaled to per SD)			1.01 (0.99-1.02)

Notes: Values show in each cell are hazard ratios and 95% confidence intervals from frailty models with N=2,753,000; Boldface indicates statistical significance (p<0.05); CBDRB-FY20-CES004-033

Table S6. Hazard ratios and 95% confidence intervals for association with cardiovascular mortality, from models using census tract estimates for healthy food retail presence and other area-based characteristics

	Minimally adjusted	Moderate adjustment	Fully Adjusted
Any supermarket or produce market present	1.08 (1.00-1.04)	1.01 (0.99-1.04)	1.03 (1.00-1.07)
Female gender	**0.45 (0.44-0.46)**	**0.43 (0.42-0.44)**	**0.43 (0.42-0.44)**
Age (rescaled to per 10 years)	**2.72 (2.69-2.74)**	**2.64 (2.62-2.66)**	**2.64 (2.62-2.66)**
Married	**0.58 (0.57-0.59)**	**0.63 (0.61-0.64)**	**0.63 (0.62-0.64)**
US born	**1.35 (1.30-1.40)**	**1.30 (1.25-1.35)**	**1.31 (1.26-1.36)**
Black race	**1.08 (1.05-1.12)**	1.01 (0.97-1.04)	**0.94 (0.91-0.98)**
Hispanic ethnicity	**0.88 (0.84-0.93)**	**0.80 (0.77-0.84)**	**0.76 (0.73-0.80)**
Educational attainment college or more		**0.65 (0.63-0.67)**	**0.66 (0.64-0.68)**
Income (rescaled, e.g., to per 10K or per SD)		**0.97 (0.97-0.98)**	**0.98 (0.98-0.98)**
Median household income (rescaled to per 10K)			**0.96 (0.96-0.97)**
Population density (residents/km^2) (rescaled to per 10K/km^2)	(shaded indicates exclude from model)		**1.12 (1.07-1.17)**
Walkable destination density (count/km^2), (rescaled to per SD)			1.00 (0.98-1.01)

Notes: Values show in each cell are hazard ratios and 95% confidence intervals from models with N=2,753,000; Boldface indicates statistical significance ($p<0.05$); CBDRB-FY20-CES004-031

Lovasi GS, *et al. BMJ Open* 2021; 11:e048390. doi: 10.1136/bmjopen-2020-048390
