# Supplementary material for: Healthy food retail availability and cardiovascular mortality in the United States: a cohort study
Source: BMJ Open. 2021 Jul 9;11(7):e048390. doi: 10.1136/bmjopen-2020-048390 (PMC8273445; doi:10.1136/bmjopen-2020-048390)
Supplement: Author's manuscript [file bmjopen-2020-048390.draft_revisions.pdf]

BMJ Open

BMJ Open is committed to open peer review. As part of this commitment we make the peer review history of every article we publish publicly available.

When an article is published we post the peer reviewers' comments and the authors' responses online. We also post the versions of the paper that were used during peer review. These are the versions that the peer review comments apply to.

The versions of the paper that follow are the versions that were submitted during the peer review process. They are not the versions of record or the final published versions. They should not be cited or distributed as the published version of this manuscript.

BMJ Open is an open access journal and the full, final, typeset and author-corrected version of record of the manuscript is available on our site with no access controls, subscription charges or pay-per-view fees (http://bmjopen.bmj.com).

If you have any questions on BMJ Open's open peer review process please email info.bmjopen@bmj.com

BMJ Open

Healthy Food Retail Availability Is Not Associated with Cardiovascular Mortality in a Representative US Sample

Journal:	*BMJ Open*
Manuscript ID	bmjopen-2020-048390
Article Type:	Original research
Date Submitted by the Author:	28-Dec-2020
Complete List of Authors:	Lovasi, Gina; Drexel University, Urban Health Collaborative, Dornsife School of Public Health<br>Johnson, Norman; Census Bureau, Center for Administrative Records and Research Applications<br>Altekruse, Sean; National Institutes of Health, National Heart Lung and Blood institute, Division of Cardiovascular Sciences<br>Hirsch, Jana ; Drexel University, Urban Health Collaborative, Dornsife School of Public Health<br>Moore, Kari; Drexel University School of Public Health, Urban Health Collaborative<br>Brown, Janene; Drexel University, Urban Health Collaborative, Dornsife School of Public Health<br>Rundle, Andrew; Columbia University, Built Environment and Health Research Group<br>Quinn, James; Columbia University, Built Environment and Health Research Group<br>Neckerman, Kathryn; Columbia University, Built Environment and Health Research Group<br>Siscovick, David; New York Academy of Medicine
Keywords:	Coronary heart disease < CARDIOLOGY, EPIDEMIOLOGY, PREVENTIVE MEDICINE, PUBLIC HEALTH

SCHOLARONE™
Manuscripts

I, the Submitting Author has the right to grant and does grant on behalf of all authors of the Work (as defined in the below author licence), an exclusive licence and/or a non-exclusive licence for contributions from authors who are: i) UK Crown employees; ii) where BMJ has agreed a CC-BY licence shall apply, and/or iii) in accordance with the terms applicable for US Federal Government officers or employees acting as part of their official duties; on a worldwide, perpetual, irrevocable, royalty-free basis to BMJ Publishing Group Ltd ("BMJ") its licensees and where the relevant Journal is co-owned by BMJ to the co-owners of the Journal, to publish the Work in this journal and any other BMJ products and to exploit all rights, as set out in our licence.

The Submitting Author accepts and understands that any supply made under these terms is made by BMJ to the Submitting Author unless you are acting as an employee on behalf of your employer or a postgraduate student of an affiliated institution which is paying any applicable article publishing charge ("APC") for Open Access articles. Where the Submitting Author wishes to make the Work available on an Open Access basis (and intends to pay the relevant APC), the terms of reuse of such Open Access shall be governed by a Creative Commons licence – details of these licences and which Creative Commons licence will apply to this Work are set out in our licence referred to above.

Other than as permitted in any relevant BMJ Author's Self Archiving Policies, I confirm this Work has not been accepted for publication elsewhere, is not being considered for publication elsewhere and does not duplicate material already published. I confirm all authors consent to publication of this Work and authorise the granting of this licence.

For peer review only

Title: Healthy Food Retail Availability Is Not Associated with Cardiovascular Mortality in a Representative US Sample

Authors: Gina S. Lovasi,[1]* Norm Johnson,[2] Sean Altekruse,[3] Jana A. Hirsch,[1] Kari Moore,[1] Janene R. Brown, [1] Andrew Rundle,[4] James Quinn,[4] Kathryn Neckerman,[4] David Siscovick[5]

[1] Drexel University, Urban Health Collaborative, Philadelphia, PA, USA

[2] Census Headquarters, Suitland, MD, USA

[3] National Institutes of Health, National Heart Lung and Blood Institute, Division of Cardiovascular Sciences, Bethesda, MD, USA

[4] Columbia University, Built Environment and Health Research Group, New York, NY, USA

[5] New York Academy of Medicine, New York City, NY, USA

*Corresponding author contact information:

Gina S. Lovasi, PhD

Urban Health Collaborative Co-Director

Dornsife Associate Professor of Urban Health

Department of Epidemiology and Biostatistics

Dornsife School of Public Health

Drexel University

3600 Market Street, Office 751

Philadelphia, PA 19104

Tel: 646-761-1362

Email: gsl45@drexel.edu

For peer review only

Keywords (MeSH): Social Determinants of Health; Residence Characteristic; Supermarkets;

Cardiovascular Diseases; Mortality

Length: 3,030 words (excluding title page, abstract, references, statements, figures and tables)

For peer review only - http://bmjopen.bmj.com/site/about/guidelines.xhtml

ABSTRACT

Introduction: Modifiable aspects of the built environment, including availability of healthy food retail, could be incorporated into population-level cardiovascular disease prevention efforts. Investigation of food source availability by type, while controlling for sociodemographic characteristics, may inform our understanding of the likely health implications of preserving or increasing food retail.

Methods: Individual-level American Community Survey data from 2008 was linked to National Death Index records through 2015, creating Mortality Disparities in American Communities (MDAC) data. Area-based data included sociodemographic and retail characteristics by ZIP code tabulation area (ZCTA). We ran proportional hazards models adjusted for potential sociodemographic and environment confounders. Results were compared across strata, using census tracts as an alternative neighborhood definition, after accounting for non-independence using frailty models, and with all-cause instead of cause-specific mortality as the outcome.

Results: Of 2,753,000 individuals age 25+ living in households with full kitchen facilities (excluding group quarters), 82% had healthy food retail (supermarket, produce market) within their ZCTA. Density of such retail was correlated with unhealthy food sources (Spearman's correlation: 0.88). Healthy food retail presence was not associated with reduced cardiovascular (HR: 1.02; 95% CI: 0.99-1.06) or all-cause mortality (HR: 1.04; 95% CI: 1.03-1.05) in fully adjusted models, or in any of the sensitivity analyses and strata considered. However, unhealthy food retail presence was associated with elevated all-cause mortality (HR: 1.14; 95% CI: 1.10-1.18).

Conclusion: Hypothesized associations of healthy food retail with cardiovascular mortality were not supported; the association of unhealthy food retail presence with mortality was not specific to cardiovascular causes.

For peer review only - http://bmjopen.bmj.com/site/about/guidelines.xhtml

ARTICLE SUMMARY

Strengths and limitations of this study

- In light of the ongoing salience of “food deserts” in policy discussions, separate consideration of healthy food store presence while controlling for potential socioeconomic confounders may reveal whether policy strategies with a focus on preserving or increasing healthy food retail are likely to improve cardiovascular outcomes.
- Data are from the Mortality Disparities in American Communities (MDAC) study, a large US-based representative sample that combines the strengths of the American Communities Survey, individual linkage to the National Death Index, and area-based characteristics.
- Our approach assessed the robustness of findings across adjustment strategies, population strata (women, men, urban residents, single-family households, and county-based groupings), analytical approaches, geographic units (postal codes or census tracts), and with variation in exposure and outcome definitions.
- Key limitations include the risks of uncontrolled confounding, exposure or outcome misclassification, and selection bias.

For peer review only

INTRODUCTION

Modifiable risk factors are associated with more than 70% of clinical cardiovascular disease (CVD),[1] the leading cause of death in the US.[2] Built environment characteristics may affect health-related behaviors that contribute to chronic disease risk, including cardiovascular morbidity and mortality,[1] potentially explaining geospatial variation in cardiovascular outcomes.[3-6]

The built environment could be improved as a component of population-level cardiovascular disease prevention efforts. Concepts such as food deserts have particular resonance in policy discussions.[7] Studies typically define food deserts through both low-income criteria and a lack of healthy food retail, as in a recent example.[8] Scarcity of healthy food retail may hinder individuals' and families' efforts to eat nutritious diets that include fresh foods.[9-13] Yet healthy food availability depends on neighborhood socioeconomic context.[10-12] An operationalization of food deserts that conflates inadequate access to healthy food retail and low area-based income can provide evidence for a policy approach that jointly tackles these challenges. However, separate consideration of healthy food store availability may better address the likely health implications of policy strategies with an exclusive focus on preserving or increasing healthy food retail.[14]

In the present study, we use food retail data linked to the Mortality Disparities in American Communities (MDAC) study. Individual and household socioeconomic data and food retail data[15] are from the 2008 American Community Survey (ACS), with outcome assessment based on National Death Index (NDI) linkage. Our analytic approach uses survival analyses, minimally adjusted for demographic characteristics, considering further adjustment for socioeconomic and contextual characteristics. We hypothesized that presence of healthy food sources near the home would be associated with lower

For peer review only - http://bmjopen.bmj.com/site/about/guidelines.xhtml

cardiovascular mortality. We consider whether food environment-mortality associations were consistent across population strata, alternative exposure and outcome specifications, and analytic approaches.

METHODS

Study sample and data linkage overview

Individual linkage of data from 2008 ACS respondents to the NDI provides a foundation for MDAC, a collaborative project of the US Census Bureau, the Centers for Disease Control and Prevention, and the National Institutes of Health.[16] The ACS sampling frame is designed to be representative across demographic categories (age, sex, race, ethnicity, and state of residence) for the US population. Sampling weights are based on annual ACS national population estimates from the US Census Bureau.

Geographic linkage used residential ZCTA and census tract. Intending to capture food environment retail reachable within a short drive, ZCTA was selected as the primary level for contextual characteristics during the MDAC proposal approval process, with a planned sensitivity analysis using census tract data. Both ZCTA and census tract geographies are systematically larger in areas of low population density.

Patient and public involvement

The analyses presented in this manuscript were investigator-initiated and did not reflect patient or public involvement, though such involvement shows promise to provide a foundation for the innovation and relevance of future inquiry.

For peer review only - http://bmjopen.bmj.com/site/about/guidelines.xhtml

Inclusion criteria

Our analytic sample was initially restricted to individuals from ACS survey households with consent for research data use (N=4,512,000; note that sample sizes in tables and to illustrate changes as inclusion criteria are applied are rounded to the thousands during disclosure proofing; CBDRB-FY20-CES004-021). We further limited to individuals for whom personal identifiers were sufficiently complete to allow linkage to NDI through December 31, 2015 (4,480,000). Due to potential differences in food acquisition, we excluded individuals residing in group quarters or in households without a full kitchen (3.8%). Linkage to ZCTA-level food environment data assembled across the continental US was completed for 4,107,000 individuals. Based on our interest in associations with cardiovascular mortality adjusted for individual socioeconomic characteristics, we restricted our analyses to adults 25+ years of age (2,923,000). Final exclusion of observations with missing covariate data resulted in an analytic sample of 2,753,000.

Geographic units and their characterization

Contextual characteristics were assembled and linked to geocoded home address data using ZCTA and census tract boundaries (TIGER Line, 2016 version of the 2010 census boundaries). The area-based characteristics considered as potential confounders, including population density and median household income, used ACS data from 2008-2012 estimates included in a harmonized Longitudinal Tract Database.[17]

For peer review only - http://bmjopen.bmj.com/site/about/guidelines.xhtml

Food retail characteristics were estimated using National Establishment Time Series (NETS) data. Steps to enhance accuracy, consistency, and replicability of our work with these data have been described elsewhere, along with the rationale and checking of our business category definitions.[15]

A combined category of healthy food retail sources was defined to include supermarkets (using chain name searches, 8-digit Standard Industrial Codes (SIC), and size thresholds: number of employees ≥ 25 or sales volume ≥ $2 million) and produce stores (fruit and vegetable market SIC codes). A secondary definition of healthy food sources included additional retail that may provide some cardioprotective benefits, but which are less common and have received limited attention in the literature (natural food, health food, and vitamin stores). For unhealthy food retail, we considered a combined category of fast food, quick service, and pizza restaurants; bakery, ice cream, coffee, and candy shops; and convenience and small grocery stores. A broadened definition of unhealthy food retail sources further included as potential sources of highly processed foods: pharmacies, gas stations, and nut stores (typically selling sweetened nuts and candy).

In addition to food retail, we consider in our maximally adjusted models control for a broader retail category labeled “walkable destinations” designed to include establishments that contribute to making pedestrian transportation attractive and feasible.[18]

We operationalized these retail categories across 1990-2014 NETS data, which contained approximately 58 million unique establishments identified by DUNS number (establishments had a mean of 1.3 distinct addresses reported over time, yielding more than 77 million records to re-geocode).[15] For alignment

For peer review only - http://bmjopen.bmj.com/site/about/guidelines.xhtml

with MDAC baseline, we use retail data from 2008 across 32,170 ZCTAs and 72,246 census tracts. Count of establishments was constructed for each retail category, dichotomized as present/absent, and used to estimate density using a land area denominator (count per km^2).

Individual demographic and household socioeconomic data

Demographic characteristics from the ACS included gender, age, marital status, nativity (US born vs other), and race/ethnicity. Socioeconomic characteristics included educational attainment, and household income. To increase interpretability, age was rescaled to 10-year increments, and income was rescaled to increments of $10,000.

Defining urban and county-based strata

Geographies were classified as urban or non-urban based on presence within an urbanized area (UAs) or urban cluster (UCs). Urbanized Areas (UAs) consist of densely developed territories that contain 50,000 or more people. Urban Clusters (UCs) consist of densely developed territories with at least 2,500 people but fewer than 50,000 people. In 2010, an estimated 81% of the US population resided in urban areas.[19]

A county-level analysis inspired by prior work on the "Eight Americas"[20] was conducted by Jahn Hakes and Sean Altekruse (personal communication, June 2, 2020), resulting in 11 strata across the continental US (additional strata defined for Alaska and Hawaii are not used here). Briefly, 39 county-level sociodemographic and climate variables (sourced from ACS and CDC WONDER[21]) were used in a principle component analysis, resulting in 6 components that were then used to assign counties into strata with ad hoc names.

For peer review only - http://bmjopen.bmj.com/site/about/guidelines.xhtml

For peer review only

All-cause and cardiovascular diseases mortality outcome definitions

The primary cardiovascular mortality outcome based on NDI (NCHS 113) included acute myocardial infarction, other acute ischemic heart diseases, atherosclerotic cardiovascular disease, atherosclerosis, and all other forms of chronic ischemic heart disease. As a secondary mortality outcome, we considered a broadened cardiometabolic mortality outcome category that includes causes of death noted above plus those related to diabetes mellitus, hypertensive heart disease, hypertensive heart and renal disease, heart failure, all other forms of heart disease, essential (primary) hypertension and hypertensive renal disease, cerebrovascular diseases, aortic aneurysm and dissection, other diseases of arteries, arterioles and capillaries, and other disorders of circulatory system. All-cause mortality was also considered, to evaluate the specificity of any associations with cause-specific mortality.

Statistical analyses

Cox proportional hazards model used as an origin the date of ACS survey response, and end of follow-up was the date of death or December 31, 2015. For cause-specific mortality analyses, death from other causes was treated as censoring. Non-independence across geographic units was accommodated through complex stratified random sample and corresponding weighting. In a sensitivity analysis, we considered frailty models accounting for clustering by county as an alternative modeling strategy.[22]

Indicators of healthy or unhealthy food retail presence were dichotomized and considered separately (not mutually adjusted due to multicollinearity concerns, based on individual-level Spearman's correlation coefficients among continuous contextual characteristics). All models minimally adjusted for

For peer review only - http://bmjopen.bmj.com/site/about/guidelines.xhtml

demographic characteristics (age, marital status, nativity, race, and ethnicity). Additional adjustment was added for educational attainment and household income, and then for contextual characteristics (area-based income, population density, and walkable destination density), both overall and for stratified analyses.

Analyses were conducted in SAS 9.4, with data storage and access restricted to devices at Census Headquarters in Suitland, MD; remote access for viewing output was provided through the Research Output Direct Access System (RODAS) system, available to GSL and JB following completion of requirements for Special Sworn Status.

RESULTS

Of 2,753,000 individuals age 25+ living in households with full kitchen facilities, 82% had healthy food retail (supermarket or produce market) within their ZCTA (Table 1). Those without healthy food retail were more likely to be married, born in the US, White, and Non-Hispanic. Those with healthy food retail had higher educational attainment and household incomes, and lived in areas with higher income, population density, walkable destination density, and unhealthy food source density.

Density of retail establishments posited to be healthy (whether defined as supermarkets alone, supermarkets and produce markets, or a more inclusive definition including natural, health, and vitamin stores) was correlated with unhealthy sources (person-level Spearman's correlation coefficients from 0.85 to 0.94). Strong correlations were also noted between food environment densities and both population density and walkable destination density (Table 2).

For peer review only - http://bmjopen.bmj.com/site/about/guidelines.xhtml

For peer review only

Presence of healthy food within the ZCTA was not associated with reduced cardiovascular mortality across adjustment strategies considered (Table 3). Similar patterns were observed in analyses that were sex stratified, restricted to urban residents, or restricted to households without multiple subfamilies (Figure S1, Tables S1, S2, S3, and S4). Conditional associations accounting for random effects by county using frailty models yielded null findings for healthy food retail, and were similar to the main analysis except that the association of population density with CVD mortality became non-significant (Table S5). A sensitivity analysis at the census tract level was similar to the main analysis; the fully adjusted hazard ratio for any supermarket or produce market with cardiovascular mortality was not statistically significant and the confidence interval excluded any meaningful protective association (HR: 1.03; 95% CI: 1.00-1.07) (Table S6). Likewise, analyses of healthy food retail presence with cardiovascular mortality did not result in a statistically significant association within any of the 11 county-based strata considered (Table 4), though we note that the strongest trend in the hypothesized direction was for the 47,000 adults in counties assigned to the Southern Rural stratum (HR: 0.74; 95% CI: 0.528-1.022). When continuous density was used instead of presence, each standard deviation of healthy food source density was associated with slightly higher cardiovascular mortality, with confidence limits that exclude any HR supportive of our hypothesized direction of association (HR: 1.03; 95% CI: 1.01 to 1.05, CBDRB-FY20-CES004-013).

We considered alternative indicators of presence of food retail by type (including both healthy and unhealthy sources) and broader cardiorespiratory and all-cause mortality outcomes (Table 5). These variations in exposure and outcome definition did not result in healthy food retail being associated with

For peer review only - http://bmjopen.bmj.com/site/about/guidelines.xhtml

reduced mortality; however, presence of healthy or unhealthy food retail were both associated with higher all-cause mortality.

DISCUSSION

While healthy food retail availability was hypothesized to be cardioprotective, we did not find support for this hypothesis in this large dataset representative of the continental US. Findings were null (or in the opposite of the hypothesized direction where statistically significant) across tiered adjustment strategies, geographic units (ZCTA or census tract), across county-based strata defined using sociodemographic and climate data, and when clustering by county was accounted for using frailty models. In our exploration of other food retail variables and outcome specifications, presence of unhealthy food retail availability was noted to be associated with higher all-cause mortality.

Our overall finding that presence of healthy food retail was not associated with cardiovascular mortality echoes a recent finding that the association of food deserts with cardiovascular outcomes may predominately reflect associations with low area-based income rather than healthy food access.[8] The national scale of the present work leaves open the possibility that our classification is not sensitive to local variation in offerings across food venues, or that features associated with healthy food retail presence (including unhealthy food sources) are obscuring a true causal association. However, recent reviews have questioned the strength of evidence linking geographically determined food environment measures to obesity,[23 24] relevant to the present work because obesity is a proposed mediator between the food environment and cardiovascular health. Gamba and colleagues[24] note the highest proportion of significant findings in the expected direction among studies examining presence of food stores (versus proximity or density), the approach we have used; however, significant findings were noted to be

For peer review only - http://bmjopen.bmj.com/site/about/guidelines.xhtml

commonly quite small and of borderline significance. Likewise, Cobb and colleagues[23] conclude that findings to date on food environment and obesity are predominately null and raise concerns about quality and consistency. Qualitative findings relevant to the food environment and food behaviors have also been reviewed, with Pitt and colleagues[25] noting salience in US contexts of food quality and affordability that varies among stores in a given category, as well as coping strategies that may importantly buffer effects of local food environment on behavior. Limitations of GIS-based measures alone, without complementary information on pricing and shopper experience, are likewise underscored in a review of the food environment by Caspi and colleagues.[26]

While our *a priori* focus was on presence of healthy food retail and cardiovascular mortality, in analyses exploring alternative exposure and outcome specification we note that all food retail measures considered were associated with higher all-cause mortality. This was especially apparent for our most inclusive definition of unhealthy food sources. The presence of fast food or other venues promoting unhealthy eating may increase risk of cardiovascular mortality, as suggested by a large study in Canada.[27] In the last three decades, there has been an expansion of fast food outlets in the US,[28 29] and an increased number of fast food restaurants in residential neighborhoods has been investigated as a determinant of cardiovascular disease outcomes and risk factors such as obesity.[1 30] Unhealthy food sources have the potential to increase consumption of highly processed and calorie dense foods.[13 31-34] Indeed, our results suggest unhealthy food store presence is associated with higher all-cause mortality.

A comment is warranted on the consistent association noted for income with cardiovascular mortality. Both household and area-based income had a small but statistically significant association with reduced cardiovascular mortality across analyses. This echoes longstanding findings of a socioeconomic gradient

For peer review only - http://bmjopen.bmj.com/site/about/guidelines.xhtml

across preventable adverse health outcomes health including cardiovascular mortality.[35] When food desert measures defined jointly by both low-income settings and a lack of healthy food retail are associated with adverse health outcomes, the interpretation may falsely implicate the food environment and misdirect attention away from tackling more fundamental causes.

While caution should be taken in interpretation of covariate coefficients, given that our analysis strategy was not optimized with those coefficients in mind,[36] future work may be warranted to understand changes in the coefficient for Black racial identity from suggesting elevated risk in minimally adjusted models to a null or protective association following adjustment for socioeconomic and contextual characteristics. Attention is needed to structural racism and racial residential segregation[37] as well as continued discourse to counter any decontextualized biological interpretation of race.[38]

Strengths and limitations

Strengths include the large, representative sample across the continental US; individual, household, and area-level sociodemographic characteristics accounted for as potential confounders; and individual linkage to the National Death Index to examine cause-specific and all-cause mortality. Further, commercially licensed point-level retail data were cleaned and coded with attention to accuracy, consistency and transparency.[15] Finally, while main analyses were pre-specified in the proposal process required for access to MDAC data, we incorporated sensitivity analyses to inform future research directions. In particular, since prior reviews have suggested effect modification by regional and population characteristics,[26] we incorporated stratified analyses and noted robustness of our null findings across strata.

For peer review only - http://bmjopen.bmj.com/site/about/guidelines.xhtml

However, several limitations should be noted. First, there may be uncontrolled confounding, as we did not have data on co-morbidities and individual-level clinical or behavioral risk factors, which can be illustrated by the example of tobacco use. Cigarette smoking is potentially associated with area-based socioeconomic status, which in turn is associated with healthy food retail. However, we do not expect that confounding by smoking accounts for the null results after controlling for socioeconomic status.

Second, error likely remains in our linkage-based outcome assessment. Specifically, under-ascertainment of mortality among Hispanic and immigrant groups may result from return to country of origin at end of life or insufficient personal identifying data for unique linkage.[39]

Third, exposure mismeasurement may arise due to residential mobility during follow-up, which is not accounted for in our assessment of food retail and other independent variables. Further, our GIS-based assessment of the food environment relied on categories of retail, without complementary measures such as food pricing. A challenge we noted was the simultaneous consideration of multiple correlated density variables.

Finally, despite attempts to leverage a sampling strategy and corresponding weights to approximate a study population representative of US adults, there may be selection bias. This could have arisen at multiple points, including when respondents decline to permit data to be used for future research. While mean household income among our study sample is higher than the corresponding area-based median household income, suggesting that higher-income households may be overrepresented, the

For peer review only - http://bmjopen.bmj.com/site/about/guidelines.xhtml

contrast may reflect the relative insensitivity of the median to inclusion of a small number of extreme high values typical of the skewed US income distribution.

Conclusion

The hypothesized association of healthy food outlet presence with reduced cardiovascular mortality was not supported in this nationally representative mortality follow-up study. This suggests that strategies aimed at addressing food deserts will miss opportunities for cardiovascular mortality improvement if the focus is exclusively on healthy food retail rather than addressing more foundational causes such as area-based income and opportunity.

For peer review only

For peer review only - http://bmjopen.bmj.com/site/about/guidelines.xhtml

Funding statement:

This work was supported by the National Institute of Aging (grants 1R01AG049970, 3R01AG049970-04S1), Commonwealth Universal Research Enhancement (C.U.R.E) program funded by the Pennsylvania Department of Health (2015 Formula award - SAP #4100072543). MDAC is supported by interagency agreements of both the National Institute on Aging and the National Heart, Lung, and Blood Institute with the U.S. Census Bureau. We also thank the Urban Health Collaborative at Drexel University, the Built Environment and Health Research Group at Columbia University, the Census Bureau, the Centers for Disease Control and Prevention and the National Institutes of Health for support in bringing together the data used in this research.

Disclaimer:

This paper is released to inform interested parties of research and to encourage discussion. Any views expressed on statistical, methodological, technical, or operational issues are those of the authors and not necessarily those of the U.S. Census Bureau. These results have been reviewed by the Census Bureau's Disclosure Review Board (DRB) to ensure that no confidential information is disclosed. The DRB release numbers are: CBDRB-FY20-CES004-013, CBDRB-FY20-CES004-021, CBDRB-FY20-022, CBDRB-FY20-CES004-030, CBDRB-FY20-CES004-031, CBDRB-FY20-CES004-033, CBDRB-FY20-CES004-043, CBDRB-FY20-CES004-038. The views expressed in this manuscript are those of the authors and do not necessarily represent the views of the National Heart, Lung, and Blood Institute; the National Institutes of Health; or the U.S. Department of Health and Human Services.

Competing interest statements:

No competing interests have been disclosed.

Data sharing:

Data sharing is restricted based on (1) terms of the licensing agreements for commercial establishment data and (2) screening of publicly released data or reports by the Census Bureau's Disclosure Review Board (CBDRB).

Contributorship statement:

The proposal, table planning, manuscript draft, and integration of coauthor comments were led by GSL. Analyses were conducted by NJ, who along with SA provided expert input into the appropriate use of and description of MDAC data. Input on methods, interpretation, and checking of table accuracy were provided by JB. Longitudinal geographic characteristics were constructed and coded with expert input on the food retail classification (JAH, KM); potential built and social environment confounders (AR, KMN); geospatial methods (JQ); and cardiovascular epidemiology (DS). All authors critically reviewed and approved of the manuscript prior to submission.

For peer review only - http://bmjopen.bmj.com/site/about/guidelines.xhtml

References:

1. Malambo P, Kengne AP, De Villiers A, et al. Built environment, selected risk factors and major cardiovascular disease outcomes: a systematic review. *PloS one* 2016;11(11):e0166846.
2. Murphy SL, Xu J, Kochanek KD, et al. Mortality in the United States, 2017. 2018
3. Diez Roux AV, Nieto FJ, Caulfield L, et al. Neighbourhood differences in diet: the Atherosclerosis Risk in Communities (ARIC) Study. *Journal of epidemiology and community health* 1999;53(1):55-63.
4. Scarborough P, Nnoaham KE, Clarke D, et al. Modelling the impact of a healthy diet on cardiovascular disease and cancer mortality. *Journal of epidemiology and community health* 2012;66(5):420-6. doi: 10.1136/jech.2010.114520 [published Online First: 2010/12/22]
5. Oyebode O, Gordon-Dseagu V, Walker A, et al. Fruit and vegetable consumption and all-cause, cancer and CVD mortality: analysis of Health Survey for England data. *Journal of epidemiology and community health* 2014;68(9):856-62. doi: 10.1136/jech-2013-203500 [published Online First: 2014/04/02]
6. Franco M, Bilal U, Diez-Roux AV. Preventing non-communicable diseases through structural changes in urban environments. *Journal of epidemiology and community health* 2015;69(6):509-11. doi: 10.1136/jech-2014-203865 [published Online First: 2014/11/15]
7. Cummins S, Macintyre S. "Food deserts"—evidence and assumption in health policy making. *BMJ* 2002;325(7361):436-38.
8. Kelli HM, Kim JH, Samman Tahhan A, et al. Living in food deserts and adverse cardiovascular outcomes in patients with cardiovascular disease. *Journal of the American Heart Association* 2019;8(4):e010694.
9. Zenk SN, Powell LM, Rimkus L, et al. Relative and absolute availability of healthier food and beverage alternatives across communities in the United States. *American journal of public health* 2014;104(11):2170-78.
10. Andreyeva T, Long MW, Brownell KD. The impact of food prices on consumption: a systematic review of research on the price elasticity of demand for food. *American journal of public health* 2010;100(2):216-22. doi: 10.2105/AJPH.2008.151415
11. Leone LA, Beth D, Ickes SB, et al. Attitudes toward fruit and vegetable consumption and farmers' market usage among low-income North Carolinians. *Journal of hunger & environmental nutrition* 2012;7(1):64-76.
12. Jetter KM, Cassady DL. The availability and cost of healthier food alternatives. *American journal of preventive medicine* 2006;30(1):38-44.
13. Lovasi GS, Hutson MA, Guerra M, et al. Built environments and obesity in disadvantaged populations. *Epidemiol Rev* 2009;31:7-20. doi: mxp005 [pii]10.1093/epirev/mxp005 [published Online First: 2009/07/11]
14. Lovasi GS, Rundle A, Bader MD, et al. Case Study 1 Healthy and Unhealthy Food Sources in New York City. *Population Health* 2018:12.
15. Hirsch JA, Moore KA, Cahill J, et al. Business Data Categorization and Refinement for Application in Longitudinal Neighborhood Health Research: a Methodology. *J Urban Health* 2020 doi: 10.1007/s11524-020-00482-2 [published Online First: 2020/10/03]
16. Altekruse SF, Cosgrove CM, Altekruse WC, et al. Socioeconomic risk factors for fatal opioid overdoses in the United States: Findings from the Mortality Disparities in American Communities Study (MDAC). *PloS one* 2020;15(1):e0227966.
17. Logan JR, Stults BJ, Xu Z. Validating population estimates for harmonized census tract data, 2000–2010. *Annals of the American Association of Geographers* 2016;106(5):1013-29.
18. Rundle AG, Chen Y, Quinn JW, et al. Development of a Neighborhood Walkability Index for Studying Neighborhood Physical Activity Contexts in Communities across the U.S. over the Past Three

For peer review only - http://bmjopen.bmj.com/site/about/guidelines.xhtml

Decades. *J Urban Health* 2019;96(4):583-90. doi: 10.1007/s11524-019-00370-4 [published Online First: 2019/06/20]

19. Ratcliffe M, Burd C, Holder K, et al. Defining rural at the US Census Bureau. *American community survey and geography brief* 2016;1(8)
20. Murray CJ, Kulkarni S, Ezzati M. Eight Americas: new perspectives on US health disparities. *American journal of preventive medicine* 2005;29(5):4-10.
21. Friede A, Reid JA, Ory HW. CDC WONDER: a comprehensive on-line public health information system of the Centers for Disease Control and Prevention. *American Journal of Public Health* 1993;83(9):1289-94.
22. Bandeen-Roche KJ, Liang K-Y. Modelling failure-time associations in data with multiple levels of clustering. *Biometrika* 1996;83(1):29-39.
23. Cobb LK, Appel LJ, Franco M, et al. The relationship of the local food environment with obesity: a systematic review of methods, study quality, and results. *Obesity* 2015;23(7):1331-44.
24. Gamba RJ, Schuchter J, Rutt C, et al. Measuring the food environment and its effects on obesity in the United States: a systematic review of methods and results. *Journal of community health* 2015;40(3):464-75.
25. Pitt E, Gallegos D, Comans T, et al. Exploring the influence of local food environments on food behaviours: a systematic review of qualitative literature. *Public health nutrition* 2017;20(13):2393-405.
26. Caspi CE, Sorensen G, Subramanian S, et al. The local food environment and diet: a systematic review. *Health & place* 2012;18(5):1172-87.
27. Daniel M, Paquet C, Auger N, et al. Association of fast-food restaurant and fruit and vegetable store densities with cardiovascular mortality in a metropolitan population. *European Journal of Epidemiology* 2010;25:711-19. doi: 10.1007/s10654-010-9499-4
28. Berger N, Kaufman TK, Bader MDM, et al. Disparities in trajectories of changes in the unhealthy food environment in New York City: A latent class growth analysis, 1990–2010. *Social Science & Medicine* 2019;234:112362. doi: 10.1016/j.socscimed.2019.112362
29. James P, Seward MW, O'Malley AJ, et al. Changes in the food environment over time: examining 40 years of data in the Framingham Heart Study. *international journal of behavioral nutrition and physical activity* 2017;14(1):1-9.
30. Jeffery RW, Baxter J, McGuire M, et al. Are fast food restaurants an environmental risk factor for obesity? *Int J Behav Nutr Phys Act* 2006;3:2. doi: 10.1186/1479-5868-3-2 [published Online First: 2006/01/27]
31. Rosenheck R. Fast food consumption and increased caloric intake: a systematic review of a trajectory towards weight gain and obesity risk. *Obesity reviews* 2008;9(6):535-47.
32. Neckerman KM. Takeaway food and health. *BMJ* 2014;348
33. Stern D, Ng SW, Popkin BM. The nutrient content of US household food purchases by store type. *American journal of preventive medicine* 2016;50(2):180-90.
34. Caspi CE, Lenk K, Pelletier JE, et al. Association between store food environment and customer purchases in small grocery stores, gas-marts, pharmacies and dollar stores. *International Journal of Behavioral Nutrition and Physical Activity* 2017;14(1):76.
35. Phelan JC, Link BG, Diez-Roux A, et al. "Fundamental causes" of social inequalities in mortality: a test of the theory. *J Health Soc Behav* 2004;45(3):265-85.
36. Westreich D, Greenland S. The table 2 fallacy: presenting and interpreting confounder and modifier coefficients. *American journal of epidemiology* 2013;177(4):292-98.
37. Williams DR, Collins C. Racial residential segregation: a fundamental cause of racial disparities in health. *Public Health Rep* 2001;116(5):404-16.

For peer review only - http://bmjopen.bmj.com/site/about/guidelines.xhtml

For peer review only

38. Ross PT, Hart-Johnson T, Santen SA, et al. Considerations for using race and ethnicity as quantitative variables in medical education research. *Perspectives on Medical Education* 2020:1-6.
39. Arias E, Eschbach K, Schauman WS, et al. The Hispanic mortality advantage and ethnic misclassification on US death certificates. *American Journal of Public Health* 2010;100(S1):S171-S77.

For peer review only

For peer review only - http://bmjopen.bmj.com/site/about/guidelines.xhtml

Table 1. Demographic, socioeconomic, and contextual characteristics among included MDAC participants by availability of healthy food retail in residential ZIP Code Tabulation Areas

	No supermarket or produce market (N=492,000*)	Any supermarket or produce market (N=2,261,000*)	Total (N=2,753,000*)
Individual demographic characteristics			
Gender, % female	52.0%	53.3%	53.1%
Age, mean (SD)	52.8 (15.7)	51.5 (16.0)	51.8 (16.0)
Marital status, % married	69.6%	63.9%	64.9%
Nativity, % US born	95.4%	85.6%	87.3%
Race/ethnicity, % Black	4.6%	9.5%	8.6%
Race/ethnicity, % White	92.0%	84.9%	85.5%
Race/ethnicity, % Hispanic	4.1%	10.6%	9.4%
Race/ethnicity, % Asian/PI	1.3%	4.6%	4.0%
Race/ethnicity, % other	2.1%	1.8%	1.9%
Socioeconomic characteristics			
Educational attainment, % college or more	21.9%	31.0%	29.3%
Annual income in $ US, mean (SD)	71,800 (76,600)	84,700 (95,300)	82,400 (92,400)
Contextual (ZCTA-based)			
Median household income, mean (SD)	55,300 (19,200)	59,800 (22,800)	59,000 (22,300)
Population density (thousands of residents/km^2), mean (SD)	24 (83)	144 (355)	123 (327)
Walkable destination density (count/km^2), mean (SD)	0.5 (3.0)	3.1 (10.0)	2.6 (9.2)
Fast food density	0.2 (1.0)	0.7 (1.8)	0.6 (1.7)
Unhealthy food sources, restricted	0.5 (2.8)	3.1 (9.7)	2.6 (8.9)
Unhealthy food sources, unrestricted	0.5 (3.2)	3.7 (11.2)	3.2 (10.3)

* Exact sample size suppressed during disclosure proofing; CBDRB-FY20-022

For peer review only

For peer review only - http://bmjopen.bmj.com/site/about/guidelines.xhtml

Table 2. Correlation matrix for contextual variables, N=2,753,000

	MHI	Pop Den	Walkable	Supermkt	Healthyv1	Healthyv2	Fast Food	Unhealthyv1	Unhealthyv2
Median household income	1								
Population density	0.20	1							
Walkable destination density	0.17	0.97	1						
Supermarket density	0.13	0.83	0.85	1					
Supermarket or produce market (Healthy v1)	0.13	0.87	0.88	0.96	1				
Healthy v1 + natural, health or vitamin stores (Healthy v2)	0.16	0.92	0.94	0.91	0.94	1			
Fast food density	0.13	0.93	0.96	0.86	0.88	0.93	1		
Fast food, quick service, pizza, convenience, small grocery, bakery, coffee shop, candy, or ice cream (Unhealthy v1)	0.14	0.97	0.99	0.85	0.88	0.94	0.97	1	
Unhealthy v1 + nut stores, pharmacies, gas stations (Unhealthy v2)	0.14	0.97	0.99	0.86	0.89	0.94	0.97	1.00*	1
	MHI	Pop Den	Walkable	Supermkt	Healthyv1	Healthyv2	Fast Food	Unhealthyv1	Unhealthyv2

Note: Values shown are Spearman rank correlation coefficients based on ZIP Code Tabulation Area (ZCTA)-based characteristics appended to individual-level records, all statistically significant with *p* <.0001; CBDRB-FY20-022

* Rounded from 0.998

For peer review only - http://bmjopen.bmj.com/site/about/guidelines.xhtml

Table 3 . Hazard ratios and 95% confidence intervals for association of healthy food retail with cardiovascular mortality, N=2,753,000 adults

	Minimally adjusted	Moderate adjustment	Fully Adjusted
Any supermarket or produce market present	0.98 (0.95-1.02)	1.03 (1.00-1.06)	1.03 (1.00-1.07)
Female gender	**0.45 (0.44-0.46)**	**0.43 (0.42-0.44)**	**0.43 (0.42-0.44)**
Age (rescaled to per 10 years)	**2.72 (2.69-2.74)**	**2.64 (2.62-2.66)**	**2.64 (2.62-2.66)**
Married	**0.58 (0.57-0.59)**	**0.63 (0.61-0.64)**	**0.63 (0.62-0.64)**
US born	**1.35 (1.30-1.40)**	**1.30 (1.25-1.35)**	**1.31 (1.26-1.36)**
Black race	**1.08 (1.05-1.12)**	1.00 (0.97-1.04)	**0.94 (0.91-0.98)**
Hispanic ethnicity	**0.89 (0.85-0.93)**	**0.80 (0.77-0.84)**	**0.76 (0.73-0.80)**
Educational attainment college or more		**0.65 (0.63-0.67)**	**0.66 (0.64-0.68)**
Income (rescaled to per 10K)		**0.97 (0.97-0.98)**	**0.98 (0.98-0.98)**
Median household income (rescaled to per 10K)			**0.96 (0.96-0.97)**
Population density (residents/km^2) (rescaled to per 10K/km^2)	(shaded indicates exclude from model for the corresponding column)		**1.12 (1.07-1.17)**
Walkable destination density (count/km^2), (rescaled to per SD)			1.00 (0.98-1.01)

Notes: Values show in each cell are hazard ratios and 95% confidence intervals from models with N=2,753,000; Boldface indicates statistical significance ($p<0.05$); CBDRB-FY20-CES004-030

For peer review only - http://bmjopen.bmj.com/site/about/guidelines.xhtml

Table 4. Variation of across county strata for association of healthy food retail with cardiovascular mortality, N=2,753,000 adults

Stratum	N	Minimally adjusted	Moderate adjustment	Fully Adjusted
North Central America	112,000	1.032 (0.891-1.194)	1.076 (0.929-1.247)	1.096 (0.942-1.274)
Mountain West America	172,000	1.016 (0.898-1.150)	1.044 (0.922-1.182)	1.018 (0.896-1.156)
Northern Tier America	330,000	0.964 (0.888-1.046)	0.992 (0.914-1.077)	1.003 (0.923-1.090)
Wealthy America	265,000	0.971 (0.856-1.110)	0.991 (0.870-1.128)	0.979 (0.859-1.116)
Middle America	322,000	1.028 (0.940-1.125)	1.076 (0.983-1.177)	1.036 (0.945-1.135)
Poor America	138,000	1.036 (0.943-1.039)	1.064 (0.968-1.169)	1.064 (0.996-1.173)
Big City America	509,000	1.025 (0.909-1.157)	1.015 (0.900-1.146)	0.984 (0.872-1.111)
Sunbelt America	132,000	0.967 (0.857-1.092)	0.992 (0.879-1.120)	0.939 (0.829-1.064)
Southern Rural America	47,000	0.741 (0.534-1.028)	0.747 (0.539-1.037)	0.735 (0.528-1.022)
Mid-Sized America	127,000	0.924 (0.784-1.090)	0.986 (0.836-1.163)	0.972 (0.823-1.148)
Beach America	211,000	0.947 (0.827-1.084)	0.957 (0.836-1.095)	0.950 (0.829-1.090)

Notes: Values show in each cell are hazard ratios and 95% confidence intervals from models; CBDRB-FY20-CES004-038

For peer review only

For peer review only - http://bmjopen.bmj.com/site/about/guidelines.xhtml

Table 5. Variation of association across alternate definitions of healthy food store availability and alternate mortality outcomes

	Cardiovascular (38,500 deaths)	Cardiometabolic (87,000 deaths)	All-cause (247,000 deaths)
**Healthy food store definition**			
Supermarket	1.01 (0.98-1.04)	**1.03 (1.01-1.05)**	**1.04 (1.03-1.05)**
Supermarket or produce market	1.02 (0.99-1.06)	**1.03 (1.01-1.05)**	**1.04 (1.03-1.05)**
Supermarket, produce market, natural/health/vitamin store	**1.05 (1.01-1.09)**	**1.05 (1.02-1.08)**	**1.06 (1.04-1.08)**
**Unhealthy food store definition**			
Fast food restaurants	1.01 (0.97-1.06)	**1.04 (1.02-1.07)**	**1.06 (1.05-1.08)**
Unhealthy food sources, restricted	1.06 (0.96-1.16)	**1.10 (1.03-1.17)**	**1.14 (1.10-1.18)**
Unhealthy food sources, unrestricted	1.03 (0.93-1.15)	1.08 (1.00-1.16)	**1.16 (1.11-1.21)**

Notes: Values show in each cell are hazard ratios and 95% confidence intervals from fully adjusted models, CBDRB-FY20-CES004-043

For peer review only

TITLE

Healthy Food Retail Availability Is Not Associated with Cardiovascular Mortality in a Representative US Sample

INTRODUCTION TO SUPPLEMENTAL TABLES AND FIGURE

Sex stratified analyses (Tables S1 and S2) and analyses restricted to urban residents (Table S3) and households with no more than one subfamily (Table S4) follow the format of Table 3, and Figure S1 depicts at a glance how these compare to the main analysis finding.

Also following a format parallel to Table 3, the following tables show results from frailty analyses to account for clustering by county (S5) and using census tract data instead of ZCTA data (S6).

For peer review only

Figure S1. Hazard ratios and 95% confidence intervals for association between healthy food availability at the ZCTA level and cardiovascular mortality, across adjustment and subgroups

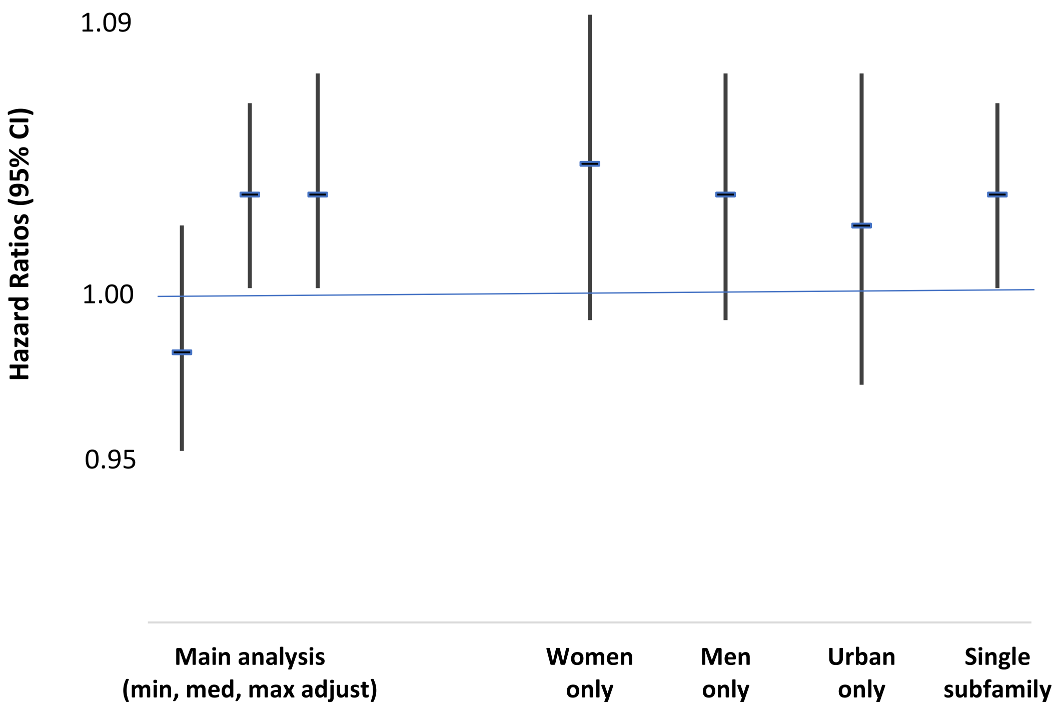

Notes: Values show are hazard ratios and 95% confidence intervals from models of healthy food retail presence with cardiovascular mortality; N=2,753,000 for main analysis, and the N is reduced for maximally adjusted stratum-specific models (1,461,000 among women, 1,292,000 among men, 1,911,000 among urban residents, and 2,711,000 among single subfamily households); CBDRB-FY20-CES004-030

review only

Table S1. Hazard ratios and 95% confidence intervals for association with cardiovascular mortality among women

	Minimally adjusted	Moderate adjustment	Fully Adjusted
Any supermarket or produce market present	1.01 (0.99-1.06)	1.04 (0.99-1.09)	1.04 (0.99-1.09)
Age (rescaled to per 10 years)	**2.93 (2.89-2.97)**	**2.84 (2.81-2.88)**	**2.84 (2.81-2.88)**
Married	**0.62 (0.60-0.65)**	**0.66 (0.64-0.69)**	**0.67 (0.64-0.69)**
US born	**1.24 (1.18-1.31)**	**1.20 (1.13-1.27)**	**1.22 (1.15-1.29)**
Black race	**1.12 (1.06-1.17)**	**1.07 (1.01-1.12)**	0.98 (0.93-1.04)
Hispanic ethnicity	**0.91 (0.84-0.97)**	**0.85 (0.79-0.91)**	**0.79 (0.74-0.85)**
Educational attainment college or more		**0.62 (0.59-0.66)**	**0.63 (0.60-0.67)**
Income (rescaled to per 10K)		**0.98 (0.97-0.98)**	**0.98 (0.98-0.98)**
Median household income (rescaled to per 10K)			**0.96 (0.95-0.97)**
Population density (residents/km^2) (rescaled to per 10K/km^2)	(shaded indicates exclude from model)		**1.20 (1.12-1.29)**
Walkable destination density (count/km^2), (rescaled to per SD)			0.99 (0.96-1.01)

Notes: Values show in each cell are hazard ratios and 95% confidence intervals from models with N=1,461,000 women; Boldface indicates statistical significance ($p<0.05$); CBDRB-FY20-CES004-030

Table S2. Hazard ratios and 95% confidence intervals for association with cardiovascular mortality among men

	Minimally adjusted	Moderate adjustment	Fully Adjusted
Any supermarket or produce market present	0.97 (0.93-1.01)	1.03 (0.98-1.07)	1.03 (0.99-1.07)
Age (rescaled to per 10 years)	**2.59 (2.57-2.62)**	**2.52 (2.49-2.54)**	**2.52 (2.49-2.55)**
Married	**0.58 (0.56-0.60)**	**0.63 (0.61-0.65)**	**0.63 (0.61-0.65)**
US born	**1.14 (1.37-1.52)**	**1.40 (1.33-1.47)**	**1.39 (1.32-1.47)**
Black race	**1.07 (1.02-1.12)**	0.96 (0.92-1.01)	**0.92 (0.87-0.96)**
Hispanic ethnicity	**0.87 (0.82-0.93)**	**0.77 (0.73-0.82)**	**0.75 (0.70-0.79)**
Educational attainment college or more		**0.67 (0.65-0.70)**	**0.69 (0.66-0.72)**
Income (rescaled to per 10K)		**0.97 (0.97-0.97)**	**0.97 (0.97-0.98)**
Median household income (rescaled to per 10K)			**0.96 (0.96-0.97)**
Population density (residents/km^2) (rescaled to per 10K/km^2)	(shaded indicates exclude from model)		1.04 (0.97-1.11)
Walkable destination density (count/km^2), (rescaled to per SD)			1.01 (0.99-1.03)

Notes: Values show in each cell are hazard ratios and 95% confidence intervals from models with N=1,292,000 men; Boldface indicates statistical significance (p<0.05); CBDRB-FY20-CES004-030

For peer review only

Table S3. Hazard ratios and 95% confidence intervals for association with cardiovascular mortality among urban residents

	Minimally adjusted	Moderate adjustment	Fully Adjusted
Any supermarket or produce market present	1.02 (0.97-1.08)	1.03 (0.98-1.09)	1.02 (0.97-1.07)
Female gender	**0.45 (0.44-0.46)**	**0.43 (0.42-0.44)**	**0.43 (0.42-0.45)**
Age (rescaled to per 10 years)	**2.73 (2.70-2.75)**	**2.65 (2.63-2.67)**	**2.65 (2.63-2.67)**
Married	**0.58 (0.57-0.60)**	**0.64 (0.62-0.65)**	**0.64 (0.62-0.66)**
US born	**1.35 (1.30-1.40)**	**1.32 (1.27-1.37)**	**1.33 (1.28-1.38)**
Black race	**1.11 (1.07-1.16)**	1.03 (0.99-1.07)	**0.96 (0.92-1.00)**
Hispanic ethnicity	**0.90 (0.85-0.94)**	**0.81 (0.77-0.85)**	**0.77 (0.73-0.81)**
Educational attainment college or more		**0.67 (0.64-0.69)**	**0.68 (0.66-0.70)**
Income (rescaled, e.g., to per 10K or per SD)		**0.97 (0.97-0.98)**	**0.98 (0.98-0.98)**
Median household income (rescaled to per 10K)			**0.96 (0.96-0.97)**
Population density (residents/km^2) (rescaled to per 10K/km^2)	(shaded indicates exclude from model)		**1.11 (1.06-1.17)**
Walkable destination density (count/km^2), (rescaled to per SD)			1.00 (0.98-1.01)

Notes: Values show in each cell are hazard ratios and 95% confidence intervals from models with N=1,911,000 urban residents; Urban was defined by the Census Bureau, based on whether the geography was within an urbanized area or urban cluster; Boldface indicates statistical significance ($p<0.05$); CBDRB-FY20-CES004-030

For peer review

Table S4. Hazard ratios and 95% confidence intervals for association with cardiovascular mortality among single family households

	Minimally adjusted	Moderate adjustment	Fully Adjusted
Any supermarket or produce market present	0.98 (0.95-1.01)	1.03 (1.00-1.06)	1.03 (1.00-1.06)
Female gender	**0.44 (0.43-0.46)**	**0.43 (0.42-0.44)**	**0.43 (0.42-0.44)**
Age (rescaled to per 10 years)	**2.72 (2.69-2.74)**	**2.63 (2.61-2.66)**	**2.64 (2.61-2.66)**
Married	**0.57 (0.56-0.59)**	**0.62 (0.61-0.64)**	**0.63 (0.61-0.64)**
US born	**1.34 (1.29-1.39)**	**1.30 (1.25-1.35)**	**1.30 (1.25-1.36)**
Black race	**1.09 (1.05-1.13)**	1.00 (0.97-1.04)	**0.94 (0.91-0.98)**
Hispanic ethnicity	**0.89 (0.85-0.93)**	**0.81 (0.77-0.85)**	**0.77 (0.73-0.81)**
Educational attainment college or more		**0.65 (0.63-0.67)**	**0.67 (0.65-0.69)**
Income (rescaled, e.g., to per 10K or per SD)		**0.97 (0.97-0.98)**	**0.98 (0.97-0.98)**
Median household income (rescaled to per 10K)			**0.96 (0.96-0.97)**
Population density (residents/km^2) (rescaled to per 10K/km^2)	(shaded indicates exclude from model)		**1.12 (1.07-1.17)**
Walkable destination density (count/km^2), (rescaled to per SD)			1.00 (0.98-1.01)

Notes: Values show in each cell are hazard ratios and 95% confidence intervals from models with N=2,711,000 in households with no more than one subfamily; Boldface indicates statistical significance ($p<0.05$); CBDRB-FY20-CES004-030

Table S5. Hazard ratios and 95% confidence intervals for association with cardiovascular mortality, conditional estimates from frailty models accounting for clustering by county

	Minimally adjusted	Moderate adjustment	Fully Adjusted
Any supermarket or produce market present	0.99 (0.96-1.03)	1.02 (0.98-1.05)	1.02 (0.99-1.05)
Female gender	**0.44 (0.43-0.45)**	**0.43 (0.42-0.44)**	**0.43 (0.42-0.44)**
Age (rescaled to per 10 years)	**2.71 (2.69-2.73)**	**2.63 (2.61-2.65)**	**2.63 (2.61-2.66)**
Married	**0.58 (0.57-0.59)**	**0.63 (0.61-0.64)**	**0.63 (0.62-0.65)**
US born	**1.38 (1.33-1.44)**	**1.38 (1.33-1.44)**	**1.38 (1.32-1.43)**
Black race	**1.05 (1.01-1.09)**	0.97 (0.93-1.01)	**0.92 (0.89-0.96)**
Hispanic ethnicity	**0.83 (0.79-0.87)**	**0.75 (0.71-0.78)**	**0.72 (0.69-0.76)**
Educational attainment college or more		**0.66 (0.64-0.68)**	**0.67 (0.65-0.69)**
Income (rescaled, e.g., to per 10K or per SD)		**0.97 (0.97-0.98)**	**0.98 (0.97-0.98)**
Median household income (rescaled to per 10K)			**0.96 (0.95-0.96)**
Population density (residents/km^2) (rescaled to per 10K/km^2)	(shaded indicates exclude from model)		0.95 (0.89-1.11)
Walkable destination density (count/km^2), (rescaled to per SD)			1.01 (0.99-1.02)

Notes: Values show in each cell are hazard ratios and 95% confidence intervals from frailty models with N=2,753,000; Boldface indicates statistical significance ($p<0.05$); CBDRB-FY20-CES004-033

For peer review only - http://bmjopen.bmj.com/site/about/guidelines.xhtml

Table S6. Hazard ratios and 95% confidence intervals for association with cardiovascular mortality, from models using census tract estimates for healthy food retail presence and other area-based characteristics

	**Minimally adjusted**	**Moderate adjustment**	**Fully Adjusted**
Any supermarket or produce market present	1.08 (1.00-1.04)	1.01 (0.99-1.04)	1.03 (1.00-1.07)
Female gender	**0.45 (0.44-0.46)**	**0.43 (0.42-0.44)**	**0.43 (0.42-0.44)**
Age (rescaled to per 10 years)	**2.72 (2.69-2.74)**	**2.64 (2.62-2.66)**	**2.64 (2.62-2.66)**
Married	**0.58 (0.57-0.59)**	**0.63 (0.61-0.64)**	**0.63 (0.62-0.64)**
US born	**1.35 (1.30-1.40)**	**1.30 (1.25-1.35)**	**1.31 (1.26-1.36)**
Black race	**1.08 (1.05-1.12)**	1.01 (0.97-1.04)	**0.94 (0.91-0.98)**
Hispanic ethnicity	**0.88 (0.84-0.93)**	**0.80 (0.77-0.84)**	**0.76 (0.73-0.80)**
Educational attainment college or more		**0.65 (0.63-0.67)**	**0.66 (0.64-0.68)**
Income (rescaled, e.g., to per 10K or per SD)		**0.97 (0.97-0.98)**	**0.98 (0.98-0.98)**
Median household income (rescaled to per 10K)			**0.96 (0.96-0.97)**
Population density (residents/km^2) (rescaled to per 10K/km^2)	(shaded indicates exclude from model)		**1.12 (1.07-1.17)**
Walkable destination density (count/km^2), (rescaled to per SD)			1.00 (0.98-1.01)

Notes: Values show in each cell are hazard ratios and 95% confidence intervals from models with N=2,753,000; Boldface indicates statistical significance ($p<0.05$); CBDRB-FY20-CES004-031

For peer review only

STROBE Statement—Checklist of items that should be included in reports of ***cohort studies***

	Item No	Recommendation
**Title and abstract**	1	(*a*) Indicate the study's design with a commonly used term in the title or the abstract<br>We specify that the data consists of a survey linked to subsequent death records. (p 2)
		(*b*) Provide in the abstract an informative and balanced summary of what was done and what was found<br>We have endeavoured to cautiously and clearly share the approach and main findings in our abstract. (p 2)
**Introduction**		
Background/rationale	2	Explain the scientific background and rationale for the investigation being reported<br>The introduction highlights the importance of cardiovascular disease mortality, and the relevance to ongoing policy debates to understanding whether and to what degree healthy food outlet availability is associated with mortality in this large adult sample. (p 4)
Objectives	3	State specific objectives, including any prespecified hypotheses<br>The hypothesized direction of association is stated, along with the aims to explore whether the association differs across population strata. (p 4-5)
**Methods**		
Study design	4	Present key elements of study design early in the paper<br>An overview of the data sources includes key aspects of the study design, followed by details on our inclusion criteria. (p 5)
Setting	5	Describe the setting, locations, and relevant dates, including periods of recruitment, exposure, follow-up, and data collection<br>The setting (continental US) and years corresponding to the ACS survey and exposure assessment (2008) and to the end of NCI linkage (2015) are specified. (p 6-8)
Participants	6	(*a*) Give the eligibility criteria, and the sources and methods of selection of participants. Describe methods of follow-up<br>Reasons for exclusion and approximate numbers are noted to illustrate attenuation of sample size (using rounding to meet requirements of Census Bureau disclosure proofing). The linkage-based mortality assessment is described and a reference to prior work provided. (p 5-6)
		(*b*) For matched studies, give matching criteria and number of exposed and unexposed<br>Not applicable
Variables	7	Clearly define all outcomes, exposures, predictors, potential confounders, and effect modifiers. Give diagnostic criteria, if applicable<br>Variables used and definitions are described, including attention to cause-specific mortality outcomes, classification of food retail, and the other variables used for weighting, description, adjustment, or stratification. (p 5-9)
Data sources/ measurement	8*	For each variable of interest, give sources of data and details of methods of assessment (measurement). Describe comparability of assessment methods if there is more than one group<br>Data sources and details are noted, and in particular both a reference and a brief description is used to convey how business establishment data were prepared for analysis. (p 5-9)
Bias	9	Describe any efforts to address potential sources of bias<br>Weighting is used to address potential selection bias. Adjustment and stratification are used to limit the influence of common prior causes that may distort the exposure-outcome association (confounding bias). (p 9-10)

For peer review only - http://bmjopen.bmj.com/site/about/guidelines.xhtml

For peer review only

Study size	10	Explain how the study size was arrived at<br>This is detailed under the subheading of "Inclusion criteria." (p 6)
Quantitative variables	11	Explain how quantitative variables were handled in the analyses. If applicable, describe which groupings were chosen and why<br>Dichotomization and rescaling are described. (p 8-9) The discussion section elaborates on the alignment between our any/none dichotomization of food environment variables and the prior literature. (p 12-13)
Statistical methods	12	(*a*) Describe all statistical methods, including those used to control for confounding<br>Time to event analyses and our tiered adjustment strategy to control for confounding are described under the subheading "Statistical analyses". (p 9-10)
		(*b*) Describe any methods used to examine subgroups and interactions<br>We describe variables used to define strata for effect modification analyses, including demographic, urban and county-based strata. In addition, clustering by county was considered in a sensitivity analysis using frailty models as an alternative modelling approach. (p 8-10)
		(*c*) Explain how missing data were addressed<br>A complete case approach to missing data is noted under the subheading of "Inclusion criteria." (p 6)
		(*d*) If applicable, explain how loss to follow-up was addressed<br>While not a traditional cohort study, the sample size was attenuated by both (1) a lack of consent to have data used for research in this linkage study and (2) inadequate identifying information to accurately link to death records. (p 6) This risks the introduction of selection bias not fully accounted for by weighting, noted as a study limitation. (p 15-16)
		(*e*) Describe any sensitivity analyses<br>Sensitivity analyses are described, including use of frailty models clustering by county and shifting our measurement of food environment and other contextual variables to the census tract level (vs ZIP code tabulation areas used in the main analysis). (p 5-9)
**Results**		
Participants	13*	(a) Report numbers of individuals at each stage of study—eg numbers potentially eligible, examined for eligibility, confirmed eligible, included in the study, completing follow-up, and analysed<br>For this linkage-based study, these details are provided in the methods section. (p 6)
		(b) Give reasons for non-participation at each stage<br>Attenuation of sample size as we apply inclusion criteria is illustrated in the methods section, though contact with participants was only at the time of survey response and mortality surveillance used linkage to the National Death Index. (p 6)
		(c) Consider use of a flow diagram<br>While considered, the narrative presentation was considered to be sufficient. (p 6)
Descriptive data	14*	(a) Give characteristics of study participants (eg demographic, clinical, social) and information on exposures and potential confounders<br>This is shown in Table 1. (p 22)
		(b) Indicate number of participants with missing data for each variable of interest<br>Exclusion of missing data was described in the methods section under the subheading "Inclusion criteria," with rounding to the thousands limiting the detail that can meaningfully be presented on variables for which missing data was rare. (p 6)
		(c) Summarise follow-up time (eg, average and total amount)<br>As shown in Table 5, the number of deaths observed was less than 10% of the total analytic sample. (p 26) Thus, the follow-up time was from survey administration to

For peer review only - http://bmjopen.bmj.com/site/about/guidelines.xhtml

		the end of follow-up in December 2015 for more than 90% of the included individuals. (p 5-6)
Outcome data	15*	Report numbers of outcome events or summary measures over time<br>This is shown in Table 5 for both cause-specific and all-cause mortality outcomes. (p 26)
Main results	16	(*a*) Give unadjusted estimates and, if applicable, confounder-adjusted estimates and their precision (eg, 95% confidence interval). Make clear which confounders were adjusted for and why they were included<br>Estimates (hazard ratios) and their 95% confidence intervals are presented, using the structure of the table or a footnote to clarify the adjustments included. (p 24-26) Unadjusted estimates were deemed to be less informative than minimally adjusted estimates given the strong association of demographic variables such as age with cardiovascular mortality, though a tiered adjustment strategy is used to illustrate the robustness of our null results as we add socioeconomic and contextual covariates.
		(*b*) Report category boundaries when continuous variables were categorized<br>Continuous variables were either maintained in models as continuous or dichotomized as any versus none (=0 versus >0). (p 8-9)
		(*c*) If relevant, consider translating estimates of relative risk into absolute risk for a meaningful time period<br>This was deemed unnecessary to inform interpretation of our largely null results.
Other analyses	17	Report other analyses done—e.g., analyses of subgroups and interactions, and sensitivity analyses<br>Other analyses are discussed in the last two paragraphs of the Results section, and illustrated either within the main tables or in supplementary materials. (p 11-12)
**Discussion**		
Key results	18	Summarise key results with reference to study objectives<br>Key results are summarized in the first paragraph of the Discussion section. (p 12)
Limitations	19	Discuss limitations of the study, taking into account sources of potential bias or imprecision. Discuss both direction and magnitude of any potential bias<br>Limitations are discussed under the subheading "Strengths and limitations," with attention to whether sources of bias are likely to occur and whether the magnitude would likely overturn the observed patterns and conclusions reached. (p 15)
Interpretation	20	Give a cautious overall interpretation of results considering objectives, limitations, multiplicity of analyses, results from similar studies, and other relevant evidence<br>Caution and the context of prior work are used in discussing our findings and their possible implications. (p 16)
Generalisability	21	Discuss the generalisability (external validity) of the study results<br>The geographic context within the continental US is discussed a strength (with the study design and use of weighting designed to approximate associations that would be observed in a nationally representative sample of adults). (p 14) However, selection bias and measurement challenges related to this national scope are also discussed among limitations. (p 15)
**Other information**		
Funding	22	Give the source of funding and the role of the funders for the present study and, if applicable, for the original study on which the present article is based<br>A disclaimer and acknowledgements of state and federal funding are provided. (p 17)

*Give information separately for exposed and unexposed groups.

For peer review only

Note: An Explanation and Elaboration article discusses each checklist item and gives methodological background and published examples of transparent reporting. The STROBE checklist is best used in conjunction with this article (freely available on the Web sites of PLoS Medicine at http://www.plosmedicine.org/, Annals of Internal Medicine at http://www.annals.org/, and Epidemiology at http://www.epidem.com/). Information on the STROBE Initiative is available at http://www.strobe-statement.org.

For peer review only

For peer review only - http://bmjopen.bmj.com/site/about/guidelines.xhtml

BMJ Open

Healthy Food Retail Availability and Cardiovascular Mortality Using Linked Data across the Contiguous US from the Mortality Disparities in American Communities Study

Journal:	*BMJ Open*
Manuscript ID	bmjopen-2020-048390.R1
Article Type:	Original research
Date Submitted by the Author:	10-May-2021
Complete List of Authors:	Lovasi, Gina; Drexel University, Urban Health Collaborative, Dornsife School of Public Health<br>Johnson, Norman; Census Bureau, Center for Administrative Records and Research Applications<br>Altekruse, Sean; National Institutes of Health, National Heart Lung and Blood institute, Division of Cardiovascular Sciences<br>Hirsch, Jana ; Drexel University, Urban Health Collaborative, Dornsife School of Public Health<br>Moore, Kari; Drexel University School of Public Health, Urban Health Collaborative<br>Brown, Janene; Drexel University, Urban Health Collaborative, Dornsife School of Public Health<br>Rundle, Andrew; Columbia University, Built Environment and Health Research Group<br>Quinn, James; Columbia University, Built Environment and Health Research Group<br>Neckerman, Kathryn; Columbia University, Built Environment and Health Research Group<br>Siscovick, David; New York Academy of Medicine
**Primary Subject Heading**:	Epidemiology
Secondary Subject Heading:	Cardiovascular medicine, Public health
Keywords:	Coronary heart disease < CARDIOLOGY, EPIDEMIOLOGY, PREVENTIVE MEDICINE, PUBLIC HEALTH

SCHOLARONE™
Manuscripts

I, the Submitting Author has the right to grant and does grant on behalf of all authors of the Work (as defined in the below author licence), an exclusive licence and/or a non-exclusive licence for contributions from authors who are: i) UK Crown employees; ii) where BMJ has agreed a CC-BY licence shall apply, and/or iii) in accordance with the terms applicable for US Federal Government officers or employees acting as part of their official duties; on a worldwide, perpetual, irrevocable, royalty-free basis to BMJ Publishing Group Ltd ("BMJ") its licensees and where the relevant Journal is co-owned by BMJ to the co-owners of the Journal, to publish the Work in this journal and any other BMJ products and to exploit all rights, as set out in our licence.

The Submitting Author accepts and understands that any supply made under these terms is made by BMJ to the Submitting Author unless you are acting as an employee on behalf of your employer or a postgraduate student of an affiliated institution which is paying any applicable article publishing charge ("APC") for Open Access articles. Where the Submitting Author wishes to make the Work available on an Open Access basis (and intends to pay the relevant APC), the terms of reuse of such Open Access shall be governed by a Creative Commons licence – details of these licences and which Creative Commons licence will apply to this Work are set out in our licence referred to above.

Other than as permitted in any relevant BMJ Author's Self Archiving Policies, I confirm this Work has not been accepted for publication elsewhere, is not being considered for publication elsewhere and does not duplicate material already published. I confirm all authors consent to publication of this Work and authorise the granting of this licence.

For peer review only

Title: Healthy Food Retail Availability and Cardiovascular Mortality Using Linked Data across the Contiguous US from the Mortality Disparities in American Communities Study.

Authors: Gina S. Lovasi,[1]* Norman J. Johnson,[2] Sean Altekruse,[3] Jana A. Hirsch,[1] Kari Moore,[1] Janene R. Brown, [1] Andrew Rundle,[4] James Quinn,[4] Kathryn Neckerman,[4] David Siscovick[5]

[1] Drexel University, Urban Health Collaborative, Philadelphia, PA, USA

[2] Census Headquarters, Suitland, MD, USA

[3] National Institutes of Health, National Heart Lung and Blood Institute, Division of Cardiovascular Sciences, Bethesda, MD, USA

[4] Columbia University, Built Environment and Health Research Group, New York, NY, USA

[5] New York Academy of Medicine, New York City, NY, USA

*Corresponding author contact information:

Gina S. Lovasi, PhD

Urban Health Collaborative Co-Director

Dornsife Associate Professor of Urban Health

Department of Epidemiology and Biostatistics

Dornsife School of Public Health

Drexel University

3600 Market Street, Office 751

Philadelphia, PA 19104

Tel: 646-761-1362

Email: gsl45@drexel.edu

Keywords (MeSH): Social Determinants of Health; Residence Characteristic; Supermarkets;

Cardiovascular Diseases; Mortality

Length: 3,326 words (excluding title page, abstract, references, statements, figures and tables)

For peer review only

For peer review only - http://bmjopen.bmj.com/site/about/guidelines.xhtml

ABSTRACT

Objectives: We investigated the association of healthy food retail presence and cardiovascular mortality, controlling for sociodemographic characteristics. This association could inform efforts to preserve or increase local supermarkets or produce market availability.

Design: Historical cohort study, combining Mortality Disparities in American Communities (individual-level data from 2008 American Community Survey linked to National Death Index records from 2008 to 2015) and retail establishment data.

Setting: Across the continental US area-based sociodemographic and retail characteristics were linked to residential location by ZIP code tabulation area (ZCTA). Sensitivity analyses used census tracts instead, restricted to urbanicity or county-based strata, or accounted for non-independence using frailty models.

Participants: 2,753,000 individuals age 25+ living in households with full kitchen facilities, excluding group quarters.

Primary and secondary outcome measures: Cardiovascular mortality (primary) and all-cause mortality (secondary).

Results: 82% had healthy food retail (supermarket, produce market) within their ZCTA. Density of such retail was correlated with density of unhealthy food sources (e.g., fast food, convenience store). Healthy food retail presence was not associated with reduced cardiovascular (HR: 1.02; 95% CI: 0.99-1.06) or all-cause mortality (HR: 1.04; 95% CI: 1.03-1.05) in fully adjusted models (with adjustment for gender, age, marital status, nativity, Black race, Hispanic ethnicity, educational attainment, income, median household income, population density, walkable destination density). The null finding for cardiovascular mortality was consistent across adjustment strategies including minimally adjusted models (individual demographics only), sensitivity analyses related to setting, and across gender or household type strata.

For peer review only - http://bmjopen.bmj.com/site/about/guidelines.xhtml

However, unhealthy food retail presence was associated with elevated all-cause mortality (HR: 1.14; 95% CI: 1.10-1.18).

Conclusions: In this study using food establishment locations within administrative areas across the US, the hypothesized association of healthy food retail availability with reduced cardiovascular mortality was not supported; an association of unhealthy food retail presence with higher mortality was not specific to cardiovascular causes.

ARTICLE SUMMARY

Strengths and limitations of this study

- In light of the ongoing salience of "food deserts" in policy discussions, separate consideration of healthy food store presence while controlling for potential socioeconomic confounders may reveal whether policy strategies with a focus on preserving or increasing healthy food retail are likely to improve cardiovascular outcomes.
- Data are from the Mortality Disparities in American Communities (MDAC) study, a large US-based representative sample that combines the strengths of the American Communities Survey, individual linkage to the National Death Index, and area-based characteristics.
- Our approach assessed the robustness of findings across adjustment strategies, population strata (women, men, urban residents, single-family households, and county-based groupings), analytical approaches, geographic units (postal codes or census tracts), and with variation in exposure and outcome definitions.
- Key limitations include the risks of uncontrolled confounding, exposure or outcome misclassification, and selection bias.

For peer review only - http://bmjopen.bmj.com/site/about/guidelines.xhtml

INTRODUCTION

Modifiable risk factors are associated with more than 70% of clinical cardiovascular disease (CVD),[1] the leading cause of death in the US.[2] Built environment characteristics may affect health-related behaviors that contribute to chronic disease risk, including cardiovascular morbidity and mortality,[1] potentially explaining geospatial variation in cardiovascular outcomes.[3-6]

The built environment could be improved as a component of population-level cardiovascular disease prevention efforts. Concepts such as food deserts have particular resonance in policy discussions.[7] Studies typically define food deserts through both low-income criteria and a lack of healthy food retail, as in a recent example.[8] Scarcity of healthy food retail may hinder individuals' and families' efforts to eat nutritious diets that include fresh foods.[9-13] Yet healthy food availability depends on neighborhood socioeconomic context.[10-12] An operationalization of food deserts that conflates inadequate access to healthy food retail and low area-based income can provide evidence for a policy approach that jointly tackles these challenges. However, separate consideration of healthy food store availability may better address the likely health implications of policy strategies with an exclusive focus on preserving or increasing healthy food retail.[14]

In the present study, we use food retail data linked to the Mortality Disparities in American Communities (MDAC) study. Individual and household socioeconomic data and food retail data[15] are from the 2008 American Community Survey (ACS), with outcome assessment based on National Death Index (NDI) linkage. Our analytic approach uses survival analyses, minimally adjusted for demographic characteristics, considering further adjustment for socioeconomic and contextual characteristics. We hypothesized that presence of healthy food sources in the home postal code area (operationalized using

For peer review only

For peer review only - http://bmjopen.bmj.com/site/about/guidelines.xhtml

ZIP code tabulation areas, ZCTAs) would be associated with lower cardiovascular mortality. We consider whether food environment-mortality associations were consistent across population strata, alternative exposure and outcome specifications, and analytic approaches.

METHODS

Study sample and data linkage overview

Individual linkage of data from 2008 ACS respondents to the NDI provides a foundation for MDAC, a collaborative project of the US Census Bureau, the Centers for Disease Control and Prevention, and the National Institutes of Health.[16] The ACS sampling frame is designed to be representative across demographic categories (age, sex, race, ethnicity, and state of residence) for the US population. Sampling weights are based on annual ACS national population estimates from the US Census Bureau.

Geographic linkage used residential ZCTA and census tract. Intending to capture food environment retail reachable within a short drive,[17] ZCTA was selected as the primary level for contextual characteristics during the MDAC proposal approval process, with a planned sensitivity analysis using census tract data. Both ZCTA and census tract geographies are systematically larger in areas of low population density.

Patient and public involvement

The analyses presented in this manuscript were investigator-initiated and did not reflect patient or public involvement, though such involvement shows promise to provide a foundation for the innovation and relevance of future inquiry.

For peer review only - http://bmjopen.bmj.com/site/about/guidelines.xhtml

Inclusion criteria

Our analytic sample was initially restricted to individuals from ACS survey households with consent for research data use (N=4,512,000; note that sample sizes in tables and to illustrate changes as inclusion criteria are applied are rounded to the thousands during disclosure proofing; CBDRB-FY20-CES004-021). We further limited to individuals for whom personal identifiers were sufficiently complete to allow linkage to NDI through December 31, 2015 (4,480,000). Due to potential differences in food acquisition, we excluded individuals residing in group quarters or in households without a full kitchen (3.8%). Linkage to ZCTA-level food environment data assembled across the continental US was completed for 4,107,000 individuals. Based on our interest in associations with cardiovascular mortality adjusted for individual socioeconomic characteristics, we restricted our analyses to adults 25+ years of age (2,923,000). Final exclusion of observations with missing covariate data resulted in an analytic sample of 2,753,000.

Geographic units and their characterization

Contextual characteristics were assembled and linked to geocoded home address data using ZCTA and census tract boundaries (TIGER Line, 2016 version of the 2010 census boundaries). The area-based characteristics considered as potential confounders, including population density and median household income, used ACS data from 2008-2012 estimates included in a harmonized Longitudinal Tract Database.[18]

For peer review only - http://bmjopen.bmj.com/site/about/guidelines.xhtml

Food retail characteristics were estimated using National Establishment Time Series (NETS) data. Steps to enhance accuracy, consistency, and replicability of our work with these data have been described elsewhere, along with the rationale and checking of our business category definitions.[15]

A combined category of healthy food retail sources was defined to include supermarkets (using chain name searches, 8-digit Standard Industrial Codes (SIC), and size thresholds: number of employees ≥ 25 or sales volume ≥ $2 million) and produce stores (fruit and vegetable market SIC codes). A secondary definition of healthy food sources included additional retail that may provide some cardioprotective benefits, but which are less common and have received limited attention in the literature (natural food, health food, and vitamin stores). For unhealthy food retail, we considered a combined category of fast food, quick service, and pizza restaurants; bakery, ice cream, coffee, and candy shops; and convenience and small grocery stores. A broadened definition of unhealthy food retail sources further included as potential sources of highly processed foods: pharmacies, gas stations, and nut stores (typically selling sweetened nuts and candy). While we recognize that establishments within the above categories offer items with varying nutritional value, our categorization was informed by prior literature and by the relative affordability of and salience of fresh items.

In addition to food retail, we consider in our maximally adjusted models control for a broader retail category labeled “walkable destinations” designed to include establishments that contribute to making pedestrian transportation attractive and feasible.[19]

For peer review only

For peer review only - http://bmjopen.bmj.com/site/about/guidelines.xhtml

We operationalized these retail categories across 1990-2014 NETS data, which contained approximately 58 million unique establishments identified by DUNS number (establishments had a mean of 1.3 distinct addresses reported over time, yielding more than 77 million records to re-geocode).[15] For alignment with MDAC baseline, we use retail data from 2008 across 32,170 ZCTAs and 72,246 census tracts. Count of establishments was constructed for each retail category, dichotomized as present/absent, and used to estimate density using a land area denominator (count per km^2).

Individual demographic and household socioeconomic data

Demographic characteristics from the ACS included gender, age, marital status, nativity (US born vs other), and race/ethnicity. Socioeconomic characteristics included educational attainment, and household income. To increase interpretability, age was rescaled to 10-year increments, and income was rescaled to increments of $10,000.

Defining urban and county-based strata

Residential location of each MDAC household was classified as urban if located within an urbanized area (UAs) or urban cluster (UCs). Urbanized Areas (UAs) consist of densely developed territories that contain 50,000 or more people. Urban Clusters (UCs) consist of densely developed territories with at least 2,500 people but fewer than 50,000 people. In 2010, an estimated 81% of the US population resided in urban areas.[20]

A county-level analysis inspired by prior work on the "Eight Americas"[21] was conducted by Jahn Hakes and Sean Altekruse (personal communication, June 2, 2020), resulting in 11 strata across the continental

For peer review only - http://bmjopen.bmj.com/site/about/guidelines.xhtml

US (additional strata defined for Alaska and Hawaii are not used here). Briefly, 39 county-level sociodemographic and climate variables (sourced from ACS and CDC WONDER[22]) were used in a principle component analysis, resulting in 6 components that were then used to assign counties into strata with ad hoc names (Southern Rural, North Central, Mid-Sized, Sunbelt, Poor, Mountain West, Beach, Wealthy, Middle, Northern Tier, and Big City America).

All-cause and cardiovascular diseases mortality outcome definitions

The primary cardiovascular mortality outcome based on NDI (based on 113 selected causes of death as defined by the Center for Disease Control and Prevention National Center for Health Statistics) included acute myocardial infarction, other acute ischemic heart diseases, atherosclerotic cardiovascular disease, atherosclerosis, and all other forms of chronic ischemic heart disease. As an alternative cardiovascular mortality outcome, we considered a broadened cardiometabolic mortality outcome category that includes causes of death noted above plus those related to diabetes mellitus, hypertensive heart disease, hypertensive heart and renal disease, heart failure, all other forms of heart disease, essential (primary) hypertension and hypertensive renal disease, cerebrovascular diseases, aortic aneurysm and dissection, other diseases of arteries, arterioles and capillaries, and other disorders of circulatory system. All-cause mortality was considered as a secondary outcome, used to evaluate the specificity of any associations with cause-specific mortality.

Statistical analyses

Cox proportional hazards model used as an origin the date of ACS survey response, and end of follow-up was the date of death or December 31, 2015. The proportional hazards assumption for our exposure of

For peer review only - http://bmjopen.bmj.com/site/about/guidelines.xhtml

interest was tested, with no significant violation detected (for the minimally adjusted model p = 0.45, for the moderately adjusted model p = 0.72, and for the fully adjusted model p = 0.91; CBDRB-FY21-CES004-020). For cause-specific mortality analyses, death from other causes was treated as censoring. Non-independence across geographic units was accommodated through complex stratified random sample and corresponding weighting. In a sensitivity analysis, we considered frailty models accounting for clustering by county as an alternative modeling strategy.[23]

Indicators of healthy or unhealthy food retail presence were dichotomized and considered separately (not mutually adjusted due to multicollinearity concerns, based on individual-level Spearman's correlation coefficients among continuous contextual characteristics). All models minimally adjusted for demographic characteristics (age, marital status, nativity, race, and ethnicity). Additional adjustment was added for educational attainment and household income, and then for contextual characteristics (area-based income, population density, and walkable destination density), both overall and for stratified analyses.

Analyses were conducted in SAS 9.4, with data storage and access restricted to devices at Census Headquarters in Suitland, MD; remote access for viewing output was provided through the Research Output Direct Access System (RODAS) system, available to GSL and JB following completion of requirements for Special Sworn Status.

RESULTS

For peer review only

For peer review only - http://bmjopen.bmj.com/site/about/guidelines.xhtml

Of 2,753,000 individuals age 25+ living in households with full kitchen facilities, 82% had healthy food retail (supermarket or produce market) within their ZCTA (Table 1). Those without healthy food retail were more likely to be married, born in the US, White, and Non-Hispanic. Those with healthy food retail had higher educational attainment and household incomes, and lived in areas with higher income, population density, walkable destination density, and unhealthy food source density.

Density of retail establishments posited to be healthy (whether defined as supermarkets alone, supermarkets and produce markets, or a more inclusive definition including natural, health, and vitamin stores) was correlated with unhealthy sources (person-level Spearman's correlation coefficients from 0.85 to 0.94). Strong correlations were also noted between food environment densities and both population density and walkable destination density (Table 2).

Presence of healthy food within the ZCTA was not associated with reduced cardiovascular mortality across adjustment strategies considered (Table 3). Similar patterns were observed in analyses that were sex stratified, restricted to urban residents, or restricted to households without multiple subfamilies (Figure S1, Tables S1, S2, S3, and S4). Conditional associations accounting for random effects by county using frailty models yielded null findings for healthy food retail, and were similar to the main analysis except that the association of population density with CVD mortality became non-significant (Table S5). A sensitivity analysis at the census tract level was similar to the main analysis; the fully adjusted hazard ratio for any supermarket or produce market with cardiovascular mortality was not statistically significant and the confidence interval excluded any meaningful protective association (HR: 1.03; 95% CI: 1.00-1.07) (Table S6). Likewise, analyses of healthy food retail presence with cardiovascular mortality did

For peer review only - http://bmjopen.bmj.com/site/about/guidelines.xhtml

not result in a statistically significant association within any of the 11 county-based strata considered (Table 4), though we note that the strongest trend in the hypothesized direction was for the 47,000 adults in counties assigned to the Southern Rural stratum (HR: 0.74; 95% CI: 0.528-1.022). When continuous density was used instead of presence, each standard deviation of healthy food source density was associated with slightly higher cardiovascular mortality, with confidence limits that exclude any HR supportive of our hypothesized direction of association (HR: 1.03; 95% CI: 1.01 to 1.05, CBDRB-FY20-CES004-013).

We considered alternative indicators of presence of food retail by type (including both healthy and unhealthy sources) and broader cardiorespiratory and all-cause mortality outcomes (Table 5). These variations in exposure and outcome definition did not result in healthy food retail being associated with reduced mortality; however, presence of healthy or unhealthy food retail were both associated with higher all-cause mortality.

DISCUSSION

While healthy food retail availability within the residential postal code area was hypothesized to be cardioprotective, we did not find support for this hypothesis in this large dataset representative of the continental US. Findings were null (or in the opposite of the hypothesized direction where statistically significant) across tiered adjustment strategies, geographic units (ZCTA or census tract), across county-based strata defined using sociodemographic and climate data, and when clustering by county was accounted for using frailty models. In our exploration of other food retail variables and outcome specifications, presence of unhealthy food retail availability was noted to be associated with higher all-cause mortality.

For peer review only - http://bmjopen.bmj.com/site/about/guidelines.xhtml

For peer review only

Our overall finding that presence of healthy food retail was not associated with cardiovascular mortality echoes a recent finding that the association of food deserts with cardiovascular outcomes may predominately reflect associations with low area-based income rather than healthy food access.[8] The national scope of the present work leaves open the possibility that our classification is not sensitive to local variation in offerings across food venues or that features associated with healthy food retail presence (including unhealthy food sources) are obscuring a true causal association. The administrative geographic areas used for measuring the food environment are systematically larger in areas with low population density, yet may not fully reflect typical distance traveled for food acquisition[17] or optimize the correspondence with subjective experience and proximal behavioral outcomes.[24] However, recent reviews have questioned the strength of evidence linking geographically determined food environment measures to obesity,[25 26] relevant to the present work because obesity is a proposed mediator between the food environment and cardiovascular health. Gamba and colleagues[26] note the highest proportion of significant findings in the expected direction among studies examining presence of food stores (versus proximity or density), the approach we have used; however, significant findings were noted to be commonly quite small and of borderline significance. Likewise, Cobb and colleagues[25] conclude that findings to date on food environment and obesity are predominately null and raise concerns about quality and consistency. Qualitative findings relevant to the food environment and food behaviors have also been reviewed, with Pitt and colleagues[27] noting salience in US contexts of food quality and affordability that varies among stores in a given category, as well as coping strategies that may importantly buffer effects of local food environment on behavior. Limitations of GIS-based measures alone, without complementary information on pricing and shopper experience, are likewise underscored in a review of the food environment by Caspi and colleagues.[28]

For peer review only - http://bmjopen.bmj.com/site/about/guidelines.xhtml

Nonetheless, further refinement of food environment exposure measures and investigation of associated cardiovascular morbidity and mortality may be warranted. Our analyses restricted to county-based strata across the US (Table 4) suggest such further investigation may particularly be warranted in settings across the rural southern counties. Prior reviews and workshops support the salience of food environment for obesity and cardiovascular disease prevention in such settings.[29 30]

While our *a priori* focus was on presence of healthy food retail and cardiovascular mortality, in analyses exploring alternative exposure and outcome specification we note that all food retail measures considered were associated with higher all-cause mortality. This was especially apparent for our most inclusive definition of unhealthy food sources. The presence of fast food or other venues promoting unhealthy eating may increase risk of cardiovascular mortality, as suggested by a large study in Canada.[31] In the last three decades, there has been an expansion of fast food outlets in the US,[32 33] and an increased number of fast food restaurants in residential neighborhoods has been investigated as a determinant of cardiovascular disease outcomes and risk factors such as obesity.[1 34] Unhealthy food sources have the potential to increase consumption of highly processed and calorie dense foods.[13 35-38] Indeed, our results suggest unhealthy food store presence is associated with higher all-cause mortality.

A comment is warranted on the consistent association noted for income with cardiovascular mortality. Both household and area-based income had a small but statistically significant association with reduced cardiovascular mortality across analyses. This echoes longstanding findings of a socioeconomic gradient across preventable adverse health outcomes health including cardiovascular mortality.[39] When food desert measures defined jointly by both low-income settings and a lack of healthy food retail are

For peer review only

associated with adverse health outcomes, the interpretation may falsely implicate the food environment and misdirect attention away from tackling more fundamental causes.

While caution should be taken in interpretation of covariate coefficients, given that our analysis strategy was not optimized with those coefficients in mind,[40] future work may be warranted to understand changes in the coefficient for Black racial identity from suggesting elevated risk in minimally adjusted models to a null or protective association following adjustment for socioeconomic and contextual characteristics. Attention is needed to structural racism and racial residential segregation[41] as well as continued discourse to counter any decontextualized biological interpretation of race.[42]

Strengths and limitations

Strengths include the large, representative sample across the continental US; individual, household, and area-level sociodemographic characteristics accounted for as potential confounders; and individual linkage to the National Death Index to examine cause-specific and all-cause mortality. Further, commercially licensed point-level retail data were cleaned and coded with attention to accuracy, consistency and transparency.[15] Finally, while main analyses were pre-specified in the proposal process required for access to MDAC data, we incorporated sensitivity analyses to inform future research directions. In particular, since prior reviews have suggested effect modification by regional and population characteristics,[28] we incorporated stratified analyses and noted robustness of our null findings across strata.

For peer review only - http://bmjopen.bmj.com/site/about/guidelines.xhtml

For peer review only

However, several limitations should be noted. First, there may be uncontrolled confounding, as we did not have data on co-morbidities and individual-level clinical or behavioral risk factors, which can be illustrated by the example of tobacco use. Cigarette smoking is potentially associated with area-based socioeconomic status, which in turn is associated with healthy food retail. We expect that controlling for individual and area-based socioeconomic status will minimize confounding by smoking, such that unmeasured confounding by smoking is unlikely to substantially account for the observed associations. However, these unmeasured characteristics could function as effect modifiers if, for example, medical advice while managing conditions such as diabetes alters how individuals respond to the local food environment.

Second, error likely remains in our linkage-based outcome assessment. Specifically, under-ascertainment of mortality among Hispanic and immigrant groups may result from return to country of origin at end of life or insufficient personal identifying data for unique linkage.[43]

Third, exposure mismeasurement may arise due to duration of residence prior to 2008 or residential mobility during follow-up, which is not accounted for in our assessment of food retail and other independent variables. Further, our GIS-based assessment of the food environment relied on categories of retail, without complementary measures such as food pricing. A challenge we noted was the simultaneous consideration of multiple correlated density variables.

Finally, despite attempts to leverage a sampling strategy and corresponding weights to approximate a study population representative of US adults, there may be selection bias. This could have arisen at

For peer review only - http://bmjopen.bmj.com/site/about/guidelines.xhtml

multiple points, including when respondents decline to permit data to be used for future research. While mean household income among our study sample is higher than the corresponding area-based median household income, suggesting that higher-income households may be overrepresented, the contrast may reflect the relative insensitivity of the median to inclusion of a small number of extreme high values typical of the skewed US income distribution.

Conclusion

The hypothesized association of healthy food outlet presence (based on the residential postal code area) with reduced cardiovascular mortality was not supported in this nationally representative mortality follow-up study. This suggests that strategies aimed at addressing food deserts will miss opportunities for cardiovascular mortality improvement if the focus is exclusively on healthy food retail rather than addressing more foundational causes such as area-based income and opportunity.

For peer review only

For peer review only - http://bmjopen.bmj.com/site/about/guidelines.xhtml

Funding statement:

This work was supported by the National Institute of Aging (grants 1R01AG049970, 3R01AG049970-04S1), Commonwealth Universal Research Enhancement (C.U.R.E) program funded by the Pennsylvania Department of Health (2015 Formula award - SAP #4100072543). MDAC is supported by interagency agreements of both the National Institute on Aging and the National Heart, Lung, and Blood Institute with the U.S. Census Bureau. We also thank the Urban Health Collaborative at Drexel University, the Built Environment and Health Research Group at Columbia University, the Census Bureau, the Centers for Disease Control and Prevention and the National Institutes of Health for support in bringing together the data used in this research.

Ethics statement:

Ethical oversight of the research involvement of Drexel investigators was provided by the Human Research Protection Program in the Office of Research & Innovation at Drexel University (IRB Protocol: 1612004989). The Mortality Disparities in American Communities consists of responses for the full year 2008 American Community Survey (ACS) followed by over seven years of mortality tracking. The ACS survey data are collected under privacy and confidentiality provisions of the U.S. Census Bureau (Title 13, US Federal Code). The assurance of confidentiality of Census Bureau data is provided by Title 13 of the United States Code. As such, MDAC operational procedures carefully follow the well-defined practices designed to maintain the confidentiality of personal records as required by Title 13.

These practices include the prevention of disclosure through the elimination of sparse cells in publications, the prohibited release of small-area geographical information on the MDAC public-use files, the use of an individually assigned MDAC control number to identify records instead of the use of personal identifiers for these purposes, and the restriction of persons having direct access to the MDAC database.

In circumstances where MDAC participants requested restrictions on the use of their data by outside investigators, their information was not linked to mortality data.

Disclaimer:

This paper is released to inform interested parties of research and to encourage discussion. Any views expressed on statistical, methodological, technical, or operational issues are those of the authors and not necessarily those of the U.S. Census Bureau. These results have been reviewed by the Census Bureau's Disclosure Review Board (DRB) to ensure that no confidential information is disclosed. The DRB release numbers are: CBDRB-FY20-CES004-013, CBDRB-FY20-CES004-021, CBDRB-FY20-022, CBDRB-FY20-CES004-030, CBDRB-FY20-CES004-031, CBDRB-FY20-CES004-033, CBDRB-FY20-CES004-043, CBDRB-FY20-CES004-038, CBDRB-FY21-CES004-020. The views expressed in this manuscript are those of the authors and do not necessarily represent the views of the National Heart, Lung, and Blood Institute; the National Institutes of Health; or the U.S. Department of Health and Human Services.

Competing interest statements:

For peer review only

No competing interests have been disclosed.

Data sharing:

Data sharing is restricted based on (1) terms of the licensing agreements for commercial establishment data and (2) screening of publicly released data or reports by the Census Bureau's Disclosure Review Board (CBDRB). Researchers interested to use the MDAC data can request access using a proposal-based process, described at https://www.census.gov/topics/research/mdac.html.

Contributorship statement:

The proposal, table planning, manuscript draft, and integration of coauthor comments were led by GL. Analyses were conducted by NJ, who along with SA provided expert input into the appropriate use of and description of MDAC data. Input on methods, interpretation, and checking of table accuracy were provided by JB. Longitudinal geographic characteristics were constructed and coded with expert input on the food retail classification (JH, KM); potential built and social environment confounders (AR, KN); geospatial methods (JQ); and cardiovascular epidemiology (DS). All authors critically reviewed and approved of the manuscript prior to submission.

For peer review only

For peer review only - http://bmjopen.bmj.com/site/about/guidelines.xhtml

References:

1. Malambo P, Kengne AP, De Villiers A, et al. Built environment, selected risk factors and major cardiovascular disease outcomes: a systematic review. *PloS one* 2016;11(11):e0166846.
2. Murphy SL, Xu J, Kochanek KD, et al. Mortality in the United States, 2017. 2018
3. Diez Roux AV, Nieto FJ, Caulfield L, et al. Neighbourhood differences in diet: the Atherosclerosis Risk in Communities (ARIC) Study. *Journal of epidemiology and community health* 1999;53(1):55-63.
4. Scarborough P, Nnoaham KE, Clarke D, et al. Modelling the impact of a healthy diet on cardiovascular disease and cancer mortality. *Journal of epidemiology and community health* 2012;66(5):420-6. doi: 10.1136/jech.2010.114520 [published Online First: 2010/12/22]
5. Oyebode O, Gordon-Dseagu V, Walker A, et al. Fruit and vegetable consumption and all-cause, cancer and CVD mortality: analysis of Health Survey for England data. *Journal of epidemiology and community health* 2014;68(9):856-62. doi: 10.1136/jech-2013-203500 [published Online First: 2014/04/02]
6. Franco M, Bilal U, Diez-Roux AV. Preventing non-communicable diseases through structural changes in urban environments. *Journal of epidemiology and community health* 2015;69(6):509-11. doi: 10.1136/jech-2014-203865 [published Online First: 2014/11/15]
7. Cummins S, Macintyre S. "Food deserts"—evidence and assumption in health policy making. *BMJ* 2002;325(7361):436-38.
8. Kelli HM, Kim JH, Samman Tahhan A, et al. Living in food deserts and adverse cardiovascular outcomes in patients with cardiovascular disease. *Journal of the American Heart Association* 2019;8(4):e010694.
9. Zenk SN, Powell LM, Rimkus L, et al. Relative and absolute availability of healthier food and beverage alternatives across communities in the United States. *American journal of public health* 2014;104(11):2170-78.
10. Andreyeva T, Long MW, Brownell KD. The impact of food prices on consumption: a systematic review of research on the price elasticity of demand for food. *American journal of public health* 2010;100(2):216-22. doi: 10.2105/AJPH.2008.151415
11. Leone LA, Beth D, Ickes SB, et al. Attitudes toward fruit and vegetable consumption and farmers' market usage among low-income North Carolinians. *Journal of hunger & environmental nutrition* 2012;7(1):64-76.
12. Jetter KM, Cassady DL. The availability and cost of healthier food alternatives. *American journal of preventive medicine* 2006;30(1):38-44.
13. Lovasi GS, Hutson MA, Guerra M, et al. Built environments and obesity in disadvantaged populations. *Epidemiol Rev* 2009;31:7-20. doi: mxp005 [pii]10.1093/epirev/mxp005 [published Online First: 2009/07/11]
14. Lovasi GS, Rundle A, Bader MD, et al. Case Study 1 Healthy and Unhealthy Food Sources in New York City. *Population Health* 2018:12.
15. Hirsch JA, Moore KA, Cahill J, et al. Business Data Categorization and Refinement for Application in Longitudinal Neighborhood Health Research: a Methodology. *J Urban Health* 2020 doi: 10.1007/s11524-020-00482-2 [published Online First: 2020/10/03]
16. Altekruse SF, Cosgrove CM, Altekruse WC, et al. Socioeconomic risk factors for fatal opioid overdoses in the United States: Findings from the Mortality Disparities in American Communities Study (MDAC). *PloS one* 2020;15(1):e0227966.
17. Ver Ploeg M, Mancino L, Todd JE, et al. Where do Americans usually shop for food and how do they travel to get there? Initial findings from the National Household Food Acquisition and Purchase Survey, 2015.

For peer review only - http://bmjopen.bmj.com/site/about/guidelines.xhtml

18. Logan JR, Stults BJ, Xu Z. Validating population estimates for harmonized census tract data, 2000–2010. *Annals of the American Association of Geographers* 2016;106(5):1013-29.
19. Rundle AG, Chen Y, Quinn JW, et al. Development of a Neighborhood Walkability Index for Studying Neighborhood Physical Activity Contexts in Communities across the U.S. over the Past Three Decades. *J Urban Health* 2019;96(4):583-90. doi: 10.1007/s11524-019-00370-4 [published Online First: 2019/06/20]
20. Ratcliffe M, Burd C, Holder K, et al. Defining rural at the US Census Bureau. *American community survey and geography brief* 2016;1(8)
21. Murray CJ, Kulkarni S, Ezzati M. Eight Americas: new perspectives on US health disparities. *American journal of preventive medicine* 2005;29(5):4-10.
22. Friede A, Reid JA, Ory HW. CDC WONDER: a comprehensive on-line public health information system of the Centers for Disease Control and Prevention. *American Journal of Public Health* 1993;83(9):1289-94.
23. Bandeen-Roche KJ, Liang K-Y. Modelling failure-time associations in data with multiple levels of clustering. *Biometrika* 1996;83(1):29-39.
24. Hirsch JA, Hillier A. Exploring the role of the food environment on food shopping patterns in Philadelphia, PA, USA: a semiquantitative comparison of two matched neighborhood groups. *International journal of environmental research and public health* 2013;10(1):295-313.
25. Cobb LK, Appel LJ, Franco M, et al. The relationship of the local food environment with obesity: a systematic review of methods, study quality, and results. *Obesity* 2015;23(7):1331-44.
26. Gamba RJ, Schuchter J, Rutt C, et al. Measuring the food environment and its effects on obesity in the United States: a systematic review of methods and results. *Journal of community health* 2015;40(3):464-75.
27. Pitt E, Gallegos D, Comans T, et al. Exploring the influence of local food environments on food behaviours: a systematic review of qualitative literature. *Public health nutrition* 2017;20(13):2393-405.
28. Caspi CE, Sorensen G, Subramanian S, et al. The local food environment and diet: a systematic review. *Health & place* 2012;18(5):1172-87.
29. Melvin CL, Corbie-Smith G, Kumanyika SK, et al. Developing a research agenda for cardiovascular disease prevention in high-risk rural communities. *American journal of public health* 2013;103(6):1011-21.
30. Calancie L, Leeman J, Jilcott Pitts SB, et al. Nutrition-related policy and environmental strategies to prevent obesity in rural communities: a systematic review of the literature, 2002–2013. 2015
31. Daniel M, Paquet C, Auger N, et al. Association of fast-food restaurant and fruit and vegetable store densities with cardiovascular mortality in a metropolitan population. *European Journal of Epidemiology* 2010;25:711-19. doi: 10.1007/s10654-010-9499-4
32. Berger N, Kaufman TK, Bader MDM, et al. Disparities in trajectories of changes in the unhealthy food environment in New York City: A latent class growth analysis, 1990–2010. *Social Science & Medicine* 2019;234:112362. doi: 10.1016/j.socscimed.2019.112362
33. James P, Seward MW, O'Malley AJ, et al. Changes in the food environment over time: examining 40 years of data in the Framingham Heart Study. *international journal of behavioral nutrition and physical activity* 2017;14(1):1-9.
34. Jeffery RW, Baxter J, McGuire M, et al. Are fast food restaurants an environmental risk factor for obesity? *Int J Behav Nutr Phys Act* 2006;3:2. doi: 10.1186/1479-5868-3-2 [published Online First: 2006/01/27]
35. Rosenheck R. Fast food consumption and increased caloric intake: a systematic review of a trajectory towards weight gain and obesity risk. *Obesity reviews* 2008;9(6):535-47.
36. Neckerman KM. Takeaway food and health. *BMJ* 2014;348

For peer review only - http://bmjopen.bmj.com/site/about/guidelines.xhtml

37. Stern D, Ng SW, Popkin BM. The nutrient content of US household food purchases by store type. *American journal of preventive medicine* 2016;50(2):180-90.
38. Caspi CE, Lenk K, Pelletier JE, et al. Association between store food environment and customer purchases in small grocery stores, gas-marts, pharmacies and dollar stores. *International Journal of Behavioral Nutrition and Physical Activity* 2017;14(1):76.
39. Phelan JC, Link BG, Diez-Roux A, et al. "Fundamental causes" of social inequalities in mortality: a test of the theory. *J Health Soc Behav* 2004;45(3):265-85.
40. Westreich D, Greenland S. The table 2 fallacy: presenting and interpreting confounder and modifier coefficients. *American journal of epidemiology* 2013;177(4):292-98.
41. Williams DR, Collins C. Racial residential segregation: a fundamental cause of racial disparities in health. *Public Health Rep* 2001;116(5):404-16.
42. Ross PT, Hart-Johnson T, Santen SA, et al. Considerations for using race and ethnicity as quantitative variables in medical education research. *Perspectives on Medical Education* 2020:1-6.
43. Arias E, Eschbach K, Schauman WS, et al. The Hispanic mortality advantage and ethnic misclassification on US death certificates. *American Journal of Public Health* 2010;100(S1):S171-S77.

For peer review only

For peer review only - http://bmjopen.bmj.com/site/about/guidelines.xhtml

Table 1. Demographic, socioeconomic, and contextual characteristics among included MDAC participants by availability of healthy food retail in residential ZIP Code Tabulation Areas

	No supermarket or produce market (N=492,000*)	Any supermarket or produce market (N=2,261,000*)	Total (N=2,753,000*)
Individual demographic characteristics			
Gender, % female	52.0%	53.3%	53.1%
Age, mean (SD)	52.8 (15.7)	51.5 (16.0)	51.8 (16.0)
Marital status, % married	69.6%	63.9%	64.9%
Nativity, % US born	95.4%	85.6%	87.3%
Race/ethnicity, % Black	4.6%	9.5%	8.6%
Race/ethnicity, % White	92.0%	84.9%	85.5%
Race/ethnicity, % Hispanic	4.1%	10.6%	9.4%
Race/ethnicity, % Asian/PI	1.3%	4.6%	4.0%
Race/ethnicity, % other	2.1%	1.8%	1.9%
Socioeconomic characteristics			
Educational attainment, % college or more	21.9%	31.0%	29.3%
Annual income in $ US, mean (SD)	71,800 (76,600)	84,700 (95,300)	82,400 (92,400)
Contextual (ZCTA-based)			
Median household income, mean (SD)	55,300 (19,200)	59,800 (22,800)	59,000 (22,300)
Population density (thousands of residents/km^2), mean (SD)	24 (83)	144 (355)	123 (327)
Walkable destination density (count/km^2), mean (SD)	0.5 (3.0)	3.1 (10.0)	2.6 (9.2)
Fast food density	0.2 (1.0)	0.7 (1.8)	0.6 (1.7)
Unhealthy food sources, restricted	0.5 (2.8)	3.1 (9.7)	2.6 (8.9)
Unhealthy food sources, unrestricted	0.5 (3.2)	3.7 (11.2)	3.2 (10.3)

* Exact sample size suppressed during disclosure proofing; CBDRB-FY20-022

For peer review only

For peer review only - http://bmjopen.bmj.com/site/about/guidelines.xhtml

Table 2. Correlation matrix for contextual variables, N=2,753,000

	MHI	Pop Den	Walkable	Supermkt	Healthyv1	Healthyv2	Fast Food	Unhealthyv1	Unhealthyv2
Median household income	1								
Population density	0.20	1							
Walkable destination density	0.17	0.97	1						
Supermarket density	0.13	0.83	0.85	1					
Supermarket or produce market (Healthy v1)	0.13	0.87	0.88	0.96	1				
Healthy v1 + natural, health or vitamin stores (Healthy v2)	0.16	0.92	0.94	0.91	0.94	1			
Fast food density	0.13	0.93	0.96	0.86	0.88	0.93	1		
Fast food, quick service, pizza, convenience, small grocery, bakery, coffee shop, candy, or ice cream (Unhealthy v1)	0.14	0.97	0.99	0.85	0.88	0.94	0.97	1	
Unhealthy v1 + nut stores, pharmacies, gas stations (Unhealthy v2)	0.14	0.97	0.99	0.86	0.89	0.94	0.97	1.00*	1
	MHI	Pop Den	Walkable	Supermkt	Healthyv1	Healthyv2	Fast Food	Unhealthyv1	Unhealthyv2

Note: Values shown are Spearman rank correlation coefficients based on ZIP Code Tabulation Area (ZCTA)-based characteristics appended to individual-level records, all statistically significant with *p* <.0001; CBDRB-FY20-022

* Rounded from 0.998

For peer review only - http://bmjopen.bmj.com/site/about/guidelines.xhtml

For peer review only

Table 3 . Hazard ratios and 95% confidence intervals for association of healthy food retail with cardiovascular mortality, N=2,753,000 adults

	Minimally adjusted	Moderate adjustment	Fully Adjusted
Any supermarket or produce market present	0.98 (0.95-1.02)	1.03 (1.00-1.06)	1.03 (1.00-1.07)
Female gender	**0.45 (0.44-0.46)**	**0.43 (0.42-0.44)**	**0.43 (0.42-0.44)**
Age (rescaled to per 10 years)	**2.72 (2.69-2.74)**	**2.64 (2.62-2.66)**	**2.64 (2.62-2.66)**
Married	**0.58 (0.57-0.59)**	**0.63 (0.61-0.64)**	**0.63 (0.62-0.64)**
US born	**1.35 (1.30-1.40)**	**1.30 (1.25-1.35)**	**1.31 (1.26-1.36)**
Black race	**1.08 (1.05-1.12)**	1.00 (0.97-1.04)	**0.94 (0.91-0.98)**
Hispanic ethnicity	**0.89 (0.85-0.93)**	**0.80 (0.77-0.84)**	**0.76 (0.73-0.80)**
Educational attainment college or more		**0.65 (0.63-0.67)**	**0.66 (0.64-0.68)**
Income (rescaled to per 10K)		**0.97 (0.97-0.98)**	**0.98 (0.98-0.98)**
Median household income (rescaled to per 10K)			**0.96 (0.96-0.97)**
Population density (residents/km²) (rescaled to per 10K/km²)	(shaded indicates exclude from model for the corresponding column)		**1.12 (1.07-1.17)**
Walkable destination density (count/km²), (rescaled to per SD)			1.00 (0.98-1.01)

Notes: Values show in each cell are hazard ratios and 95% confidence intervals from models with N=2,753,000; Boldface indicates statistical significance (p<0.05); CBDRB-FY20-CES004-030

For peer review only - http://bmjopen.bmj.com/site/about/guidelines.xhtml

Table 4. Variation of across county strata for association of healthy food retail with cardiovascular mortality, N=2,753,000 adults

Stratum	N	Minimally adjusted	Moderate adjustment	Fully Adjusted
Southern Rural America	47,000	0.74 (0.53-1.03)	0.75 (0.54-1.04)	0.74 (0.53-1.022)
North Central America	112,000	1.03 (0.89-1.19)	1.08 (0.93-1.25)	1.10 (0.94-1.274)
Mid-Sized America	127,000	0.92 (0.78-1.09)	0.99 (0.84-1.16)	0.97 (0.82-1.148)
Sunbelt America	132,000	0.97 (0.86-1.09)	0.99 (0.88-1.12)	0.94 (0.83-1.064)
Poor America	138,000	1.04 (0.94-1.04)	1.06 (0.97-1.17)	1.06 (1.00-1.17)
Mountain West America	172,000	1.02 (0.90-1.15)	1.04 (0.92-1.18)	1.0 (0.90-1.16)
Beach America	211,000	0.95 (0.83-1.08)	0.96 (0.84-1.10)	0.95 (0.83-1.09)
Wealthy America	265,000	0.97 (0.86-1.11)	0.99 (0.87-1.13)	0.98 (0.86-1.12)
Middle America	322,000	1.03 (0.94-1.13)	1.08 (0.98-1.18)	1.04 (0.95-1.14)
Northern Tier America	330,000	0.96 (0.89-1.05)	0.99 (0.91-1.08)	1.00 (0.92-1.09)
Big City America	509,000	1.03 (0.91-1.16)	1.02 (0.90-1.15)	0.98 (0.87-1.11)

Notes: Values show in each cell are hazard ratios and 95% confidence intervals from models adjusted for gender, age, marital status, nativity, Black race, Hispanic ethnicity, educational attainment, income, median household income, population density, and walkable destination density; CBDRB-FY20-CES004-038

For peer review only

Table 5. Variation of association across alternate definitions of healthy food store availability and alternate mortality outcomes

	**Cardiovascular (38,500 deaths)**	**Cardiometabolic (87,000 deaths)**	**All-cause (247,000 deaths)**
**Healthy food store definition**			
Supermarket	1.01 (0.98-1.04)	**1.03 (1.01-1.05)**	**1.04 (1.03-1.05)**
Supermarket or produce market	1.02 (0.99-1.06)	**1.03 (1.01-1.05)**	**1.04 (1.03-1.05)**
Supermarket, produce market, natural/health/vitamin store	**1.05 (1.01-1.09)**	**1.05 (1.02-1.08)**	**1.06 (1.04-1.08)**
**Unhealthy food store definition**			
Fast food restaurants	1.01 (0.97-1.06)	**1.04 (1.02-1.07)**	**1.06 (1.05-1.08)**
Unhealthy food sources, restricted	1.06 (0.96-1.16)	**1.10 (1.03-1.17)**	**1.14 (1.10-1.18)**
Unhealthy food sources, unrestricted	1.03 (0.93-1.15)	1.08 (1.00-1.16)	**1.16 (1.11-1.21)**

Notes: Values show in each cell are hazard ratios and 95% confidence intervals from models adjusted for gender, age, marital status, nativity, Black race, Hispanic ethnicity, educational attainment, income, median household income, population density, and walkable destination density; CBDRB-FY20-CES004-043

For peer review only - http://bmjopen.bmj.com/site/about/guidelines.xhtml

For peer review only

Healthy Food Retail Availability and Cardiovascular Mortality Using Linked Data across the Contiguous US from the Mortality Disparities in American Communities Study (Supplemental materials)

INTRODUCTION TO SUPPLEMENTAL TABLES AND FIGURE

Sex stratified analyses (Tables S1 and S2) and analyses restricted to urban residents (Table S3) and households with no more than one subfamily (Table S4) follow the format of Table 3, and Figure S1 depicts at a glance how these compare to the main analysis finding.

Also following a format parallel to Table 3, the following tables show results from frailty analyses to account for clustering by county (S5) and using census tract data instead of ZCTA data (S6).

For peer review only - http://bmjopen.bmj.com/site/about/guidelines.xhtml

Figure S1. Hazard ratios and 95% confidence intervals for association between healthy food availability at the ZCTA level and cardiovascular mortality, across adjustment and subgroups

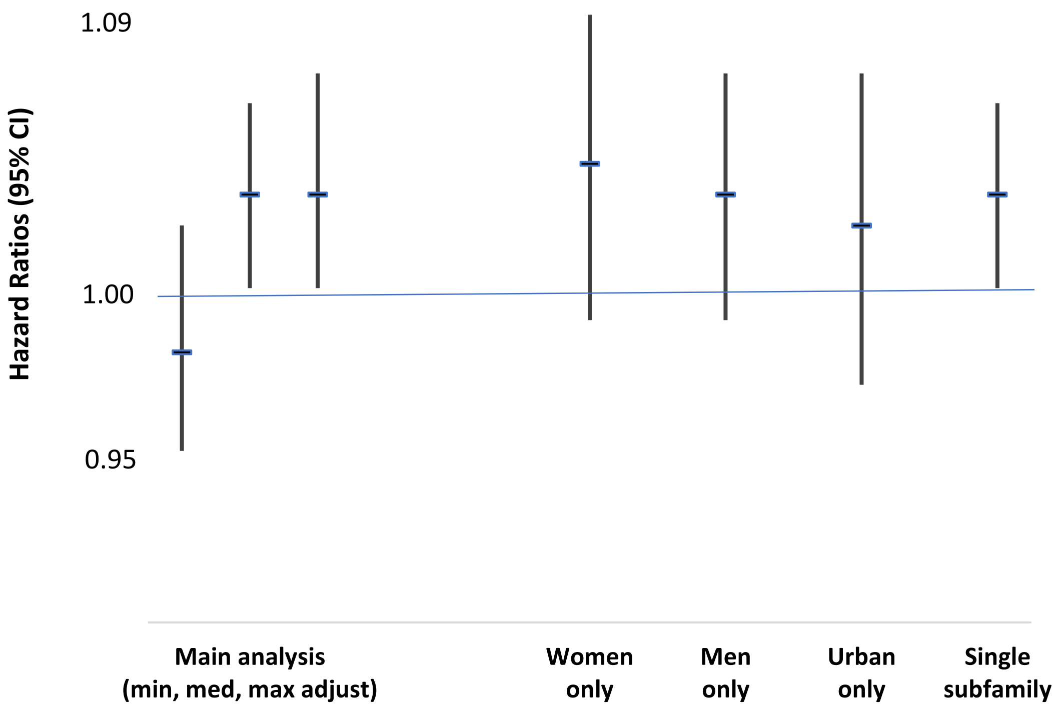

Notes: Values show are hazard ratios and 95% confidence intervals from models of healthy food retail presence with cardiovascular mortality, where "min" indicates minimally adjusted main analysis models which included gender, age, marital status, nativity, Black race, and Hispanic ethnicity; "med" indicates moderately adjusted main analysis models which included adjustment all covariates in "min" plus educational attainment and income; and "max" indicates maximally adjusted models adjusted for gender (except in gender stratified models), age, marital status, nativity, Black race, Hispanic ethnicity, educational attainment, income, median household income, population density, and walkable destination density; N=2,753,000 for main analysis, and the N is reduced for maximally adjusted stratum-specific models (1,461,000 among women, 1,292,000 among men, 1,911,000 among urban residents, and 2,711,000 among single subfamily households); CBDRB-FY20-CES004-030

For peer review only - http://bmjopen.bmj.com/site/about/guidelines.xhtml

Table S1. Hazard ratios and 95% confidence intervals for association with cardiovascular mortality among women

	**Minimally adjusted**	**Moderate adjustment**	**Fully Adjusted**
Any supermarket or produce market present	1.01 (0.99-1.06)	1.04 (0.99-1.09)	1.04 (0.99-1.09)
Age (rescaled to per 10 years)	**2.93 (2.89-2.97)**	**2.84 (2.81-2.88)**	**2.84 (2.81-2.88)**
Married	**0.62 (0.60-0.65)**	**0.66 (0.64-0.69)**	**0.67 (0.64-0.69)**
US born	**1.24 (1.18-1.31)**	**1.20 (1.13-1.27)**	**1.22 (1.15-1.29)**
Black race	**1.12 (1.06-1.17)**	**1.07 (1.01-1.12)**	0.98 (0.93-1.04)
Hispanic ethnicity	**0.91 (0.84-0.97)**	**0.85 (0.79-0.91)**	**0.79 (0.74-0.85)**
Educational attainment college or more		**0.62 (0.59-0.66)**	**0.63 (0.60-0.67)**
Income (rescaled to per 10K)		**0.98 (0.97-0.98)**	**0.98 (0.98-0.98)**
Median household income (rescaled to per 10K)			**0.96 (0.95-0.97)**
Population density (residents/km^2) (rescaled to per 10K/km^2)	(shaded indicates exclude from model)		**1.20 (1.12-1.29)**
Walkable destination density (count/km^2), (rescaled to per SD)			0.99 (0.96-1.01)

Notes: Values show in each cell are hazard ratios and 95% confidence intervals from models with N=1,461,000 women; Boldface indicates statistical significance (p<0.05); CBDRB-FY20-CES004-030

For peer review only - http://bmjopen.bmj.com/site/about/guidelines.xhtml

Table S2. Hazard ratios and 95% confidence intervals for association with cardiovascular mortality among men

	Minimally adjusted	Moderate adjustment	Fully Adjusted
Any supermarket or produce market present	0.97 (0.93-1.01)	1.03 (0.98-1.07)	1.03 (0.99-1.07)
Age (rescaled to per 10 years)	**2.59 (2.57-2.62)**	**2.52 (2.49-2.54)**	**2.52 (2.49-2.55)**
Married	**0.58 (0.56-0.60)**	**0.63 (0.61-0.65)**	**0.63 (0.61-0.65)**
US born	**1.14 (1.37-1.52)**	**1.40 (1.33-1.47)**	**1.39 (1.32-1.47)**
Black race	**1.07 (1.02-1.12)**	0.96 (0.92-1.01)	**0.92 (0.87-0.96)**
Hispanic ethnicity	**0.87 (0.82-0.93)**	**0.77 (0.73-0.82)**	**0.75 (0.70-0.79)**
Educational attainment college or more		**0.67 (0.65-0.70)**	**0.69 (0.66-0.72)**
Income (rescaled to per 10K)		**0.97 (0.97-0.97)**	**0.97 (0.97-0.98)**
Median household income (rescaled to per 10K)			**0.96 (0.96-0.97)**
Population density (residents/km^2) (rescaled to per 10K/km^2)	(shaded indicates exclude from model)		1.04 (0.97-1.11)
Walkable destination density (count/km^2), (rescaled to per SD)			1.01 (0.99-1.03)

Notes: Values show in each cell are hazard ratios and 95% confidence intervals from models with N=1,292,000 men; Boldface indicates statistical significance (p<0.05); CBDRB-FY20-CES004-030

For peer review only - http://bmjopen.bmj.com/site/about/guidelines.xhtml

Table S3. Hazard ratios and 95% confidence intervals for association with cardiovascular mortality among urban residents

	Minimally adjusted	Moderate adjustment	Fully Adjusted
Any supermarket or produce market present	1.02 (0.97-1.08)	1.03 (0.98-1.09)	1.02 (0.97-1.07)
Female gender	**0.45 (0.44-0.46)**	**0.43 (0.42-0.44)**	**0.43 (0.42-0.45)**
Age (rescaled to per 10 years)	**2.73 (2.70-2.75)**	**2.65 (2.63-2.67)**	**2.65 (2.63-2.67)**
Married	**0.58 (0.57-0.60)**	**0.64 (0.62-0.65)**	**0.64 (0.62-0.66)**
US born	**1.35 (1.30-1.40)**	**1.32 (1.27-1.37)**	**1.33 (1.28-1.38)**
Black race	**1.11 (1.07-1.16)**	1.03 (0.99-1.07)	**0.96 (0.92-1.00)**
Hispanic ethnicity	**0.90 (0.85-0.94)**	**0.81 (0.77-0.85)**	**0.77 (0.73-0.81)**
Educational attainment college or more		**0.67 (0.64-0.69)**	**0.68 (0.66-0.70)**
Income (rescaled, e.g., to per 10K or per SD)		**0.97 (0.97-0.98)**	**0.98 (0.98-0.98)**
Median household income (rescaled to per 10K)			**0.96 (0.96-0.97)**
Population density (residents/km^2) (rescaled to per 10K/km^2)	(shaded indicates exclude from model)		**1.11 (1.06-1.17)**
Walkable destination density (count/km^2), (rescaled to per SD)			1.00 (0.98-1.01)

Notes: Values show in each cell are hazard ratios and 95% confidence intervals from models with N=1,911,000 urban residents; Urban was defined by the Census Bureau, based on whether the geography was within an urbanized area or urban cluster; Boldface indicates statistical significance (p<0.05); CBDRB-FY20-CES004-030

For peer review only - http://bmjopen.bmj.com/site/about/guidelines.xhtml

Table S4. Hazard ratios and 95% confidence intervals for association with cardiovascular mortality among single family households

	Minimally adjusted	Moderate adjustment	Fully Adjusted
Any supermarket or produce market present	0.98 (0.95-1.01)	1.03 (1.00-1.06)	1.03 (1.00-1.06)
Female gender	**0.44 (0.43-0.46)**	**0.43 (0.42-0.44)**	**0.43 (0.42-0.44)**
Age (rescaled to per 10 years)	**2.72 (2.69-2.74)**	**2.63 (2.61-2.66)**	**2.64 (2.61-2.66)**
Married	**0.57 (0.56-0.59)**	**0.62 (0.61-0.64)**	**0.63 (0.61-0.64)**
US born	**1.34 (1.29-1.39)**	**1.30 (1.25-1.35)**	**1.30 (1.25-1.36)**
Black race	**1.09 (1.05-1.13)**	1.00 (0.97-1.04)	**0.94 (0.91-0.98)**
Hispanic ethnicity	**0.89 (0.85-0.93)**	**0.81 (0.77-0.85)**	**0.77 (0.73-0.81)**
Educational attainment college or more		**0.65 (0.63-0.67)**	**0.67 (0.65-0.69)**
Income (rescaled, e.g., to per 10K or per SD)		**0.97 (0.97-0.98)**	**0.98 (0.97-0.98)**
Median household income (rescaled to per 10K)			**0.96 (0.96-0.97)**
Population density (residents/km^2) (rescaled to per 10K/km^2)	(shaded indicates exclude from model)		**1.12 (1.07-1.17)**
Walkable destination density (count/km^2), (rescaled to per SD)			1.00 (0.98-1.01)

Notes: Values show in each cell are hazard ratios and 95% confidence intervals from models with N=2,711,000 in households with no more than one subfamily; Boldface indicates statistical significance ($p<0.05$); CBDRB-FY20-CES004-030

For peer review only - http://bmjopen.bmj.com/site/about/guidelines.xhtml

Table S5. Hazard ratios and 95% confidence intervals for association with cardiovascular mortality, conditional estimates from frailty models accounting for clustering by county

	Minimally adjusted	Moderate adjustment	Fully Adjusted
Any supermarket or produce market present	0.99 (0.96-1.03)	1.02 (0.98-1.05)	1.02 (0.99-1.05)
Female gender	**0.44 (0.43-0.45)**	**0.43 (0.42-0.44)**	**0.43 (0.42-0.44)**
Age (rescaled to per 10 years)	**2.71 (2.69-2.73)**	**2.63 (2.61-2.65)**	**2.63 (2.61-2.66)**
Married	**0.58 (0.57-0.59)**	**0.63 (0.61-0.64)**	**0.63 (0.62-0.65)**
US born	**1.38 (1.33-1.44)**	**1.38 (1.33-1.44)**	**1.38 (1.32-1.43)**
Black race	**1.05 (1.01-1.09)**	0.97 (0.93-1.01)	**0.92 (0.89-0.96)**
Hispanic ethnicity	**0.83 (0.79-0.87)**	**0.75 (0.71-0.78)**	**0.72 (0.69-0.76)**
Educational attainment college or more		**0.66 (0.64-0.68)**	**0.67 (0.65-0.69)**
Income (rescaled, e.g., to per 10K or per SD)		**0.97 (0.97-0.98)**	**0.98 (0.97-0.98)**
Median household income (rescaled to per 10K)			**0.96 (0.95-0.96)**
Population density (residents/km^2) (rescaled to per 10K/km^2)	(shaded indicates exclude from model)		0.95 (0.89-1.11)
Walkable destination density (count/km^2), (rescaled to per SD)			1.01 (0.99-1.02)

Notes: Values show in each cell are hazard ratios and 95% confidence intervals from frailty models with N=2,753,000; Boldface indicates statistical significance (p<0.05); CBDRB-FY20-CES004-033

For peer review only - http://bmjopen.bmj.com/site/about/guidelines.xhtml

Table S6. Hazard ratios and 95% confidence intervals for association with cardiovascular mortality, from models using census tract estimates for healthy food retail presence and other area-based characteristics

	Minimally adjusted	Moderate adjustment	Fully Adjusted
Any supermarket or produce market present	1.08 (1.00-1.04)	1.01 (0.99-1.04)	1.03 (1.00-1.07)
Female gender	**0.45 (0.44-0.46)**	**0.43 (0.42-0.44)**	**0.43 (0.42-0.44)**
Age (rescaled to per 10 years)	**2.72 (2.69-2.74)**	**2.64 (2.62-2.66)**	**2.64 (2.62-2.66)**
Married	**0.58 (0.57-0.59)**	**0.63 (0.61-0.64)**	**0.63 (0.62-0.64)**
US born	**1.35 (1.30-1.40)**	**1.30 (1.25-1.35)**	**1.31 (1.26-1.36)**
Black race	**1.08 (1.05-1.12)**	1.01 (0.97-1.04)	**0.94 (0.91-0.98)**
Hispanic ethnicity	**0.88 (0.84-0.93)**	**0.80 (0.77-0.84)**	**0.76 (0.73-0.80)**
Educational attainment college or more		**0.65 (0.63-0.67)**	**0.66 (0.64-0.68)**
Income (rescaled, e.g., to per 10K or per SD)		**0.97 (0.97-0.98)**	**0.98 (0.98-0.98)**
Median household income (rescaled to per 10K)			**0.96 (0.96-0.97)**
Population density (residents/km^2) (rescaled to per 10K/km^2)	(shaded indicates exclude from model)		**1.12 (1.07-1.17)**
Walkable destination density (count/km^2), (rescaled to per SD)			1.00 (0.98-1.01)

Notes: Values show in each cell are hazard ratios and 95% confidence intervals from models with N=2,753,000; Boldface indicates statistical significance (p<0.05); CBDRB-FY20-CES004-031

For peer review only - http://bmjopen.bmj.com/site/about/guidelines.xhtml

STROBE Statement—Checklist of items that should be included in reports of ***cohort studies***

	Item No	Recommendation
**Title and abstract**	1	(*a*) Indicate the study's design with a commonly used term in the title or the abstract<br>We specify that the data consists of a survey linked to subsequent death records. (p 2)
		(*b*) Provide in the abstract an informative and balanced summary of what was done and what was found<br>We have endeavoured to cautiously and clearly share the approach and main findings in our abstract. (p 2)
**Introduction**		
Background/rationale	2	Explain the scientific background and rationale for the investigation being reported<br>The introduction highlights the importance of cardiovascular disease mortality, and the relevance to ongoing policy debates to understanding whether and to what degree healthy food outlet availability is associated with mortality in this large adult sample. (p 4)
Objectives	3	State specific objectives, including any prespecified hypotheses<br>The hypothesized direction of association is stated, along with the aims to explore whether the association differs across population strata. (p 4-5)
**Methods**		
Study design	4	Present key elements of study design early in the paper<br>An overview of the data sources includes key aspects of the study design, followed by details on our inclusion criteria. (p 5)
Setting	5	Describe the setting, locations, and relevant dates, including periods of recruitment, exposure, follow-up, and data collection<br>The setting (continental US) and years corresponding to the ACS survey and exposure assessment (2008) and to the end of NCI linkage (2015) are specified. (p 6-8)
Participants	6	(*a*) Give the eligibility criteria, and the sources and methods of selection of participants. Describe methods of follow-up<br>Reasons for exclusion and approximate numbers are noted to illustrate attenuation of sample size (using rounding to meet requirements of Census Bureau disclosure proofing). The linkage-based mortality assessment is described and a reference to prior work provided. (p 5-6)
		(*b*) For matched studies, give matching criteria and number of exposed and unexposed<br>Not applicable
Variables	7	Clearly define all outcomes, exposures, predictors, potential confounders, and effect modifiers. Give diagnostic criteria, if applicable<br>Variables used and definitions are described, including attention to cause-specific mortality outcomes, classification of food retail, and the other variables used for weighting, description, adjustment, or stratification. (p 5-9)
Data sources/ measurement	8*	For each variable of interest, give sources of data and details of methods of assessment (measurement). Describe comparability of assessment methods if there is more than one group<br>Data sources and details are noted, and in particular both a reference and a brief description is used to convey how business establishment data were prepared for analysis. (p 5-9)
Bias	9	Describe any efforts to address potential sources of bias<br>Weighting is used to address potential selection bias. Adjustment and stratification are used to limit the influence of common prior causes that may distort the exposure-outcome association (confounding bias). (p 9-10)

For peer review only

Study size	10	Explain how the study size was arrived at<br>This is detailed under the subheading of "Inclusion criteria." (p 6)
Quantitative variables	11	Explain how quantitative variables were handled in the analyses. If applicable, describe which groupings were chosen and why<br>Dichotomization and rescaling are described. (p 8-9) The discussion section elaborates on the alignment between our any/none dichotomization of food environment variables and the prior literature. (p 12-13)
Statistical methods	12	(*a*) Describe all statistical methods, including those used to control for confounding<br>Time to event analyses and our tiered adjustment strategy to control for confounding are described under the subheading "Statistical analyses". (p 9-10)
		(*b*) Describe any methods used to examine subgroups and interactions<br>We describe variables used to define strata for effect modification analyses, including demographic, urban and county-based strata. In addition, clustering by county was considered in a sensitivity analysis using frailty models as an alternative modelling approach. (p 8-10)
		(*c*) Explain how missing data were addressed<br>A complete case approach to missing data is noted under the subheading of "Inclusion criteria." (p 6)
		(*d*) If applicable, explain how loss to follow-up was addressed<br>While not a traditional cohort study, the sample size was attenuated by both (1) a lack of consent to have data used for research in this linkage study and (2) inadequate identifying information to accurately link to death records. (p 6) This risks the introduction of selection bias not fully accounted for by weighting, noted as a study limitation. (p 15-16)
		(*e*) Describe any sensitivity analyses<br>Sensitivity analyses are described, including use of frailty models clustering by county and shifting our measurement of food environment and other contextual variables to the census tract level (vs ZIP code tabulation areas used in the main analysis). (p 5-9)
**Results**		
Participants	13*	(a) Report numbers of individuals at each stage of study—eg numbers potentially eligible, examined for eligibility, confirmed eligible, included in the study, completing follow-up, and analysed<br>For this linkage-based study, these details are provided in the methods section. (p 6)
		(b) Give reasons for non-participation at each stage<br>Attenuation of sample size as we apply inclusion criteria is illustrated in the methods section, though contact with participants was only at the time of survey response and mortality surveillance used linkage to the National Death Index. (p 6)
		(c) Consider use of a flow diagram<br>While considered, the narrative presentation was considered to be sufficient. (p 6)
Descriptive data	14*	(a) Give characteristics of study participants (eg demographic, clinical, social) and information on exposures and potential confounders<br>This is shown in Table 1. (p 22)
		(b) Indicate number of participants with missing data for each variable of interest<br>Exclusion of missing data was described in the methods section under the subheading "Inclusion criteria," with rounding to the thousands limiting the detail that can meaningfully be presented on variables for which missing data was rare. (p 6)
		(c) Summarise follow-up time (eg, average and total amount)<br>As shown in Table 5, the number of deaths observed was less than 10% of the total analytic sample. (p 26) Thus, the follow-up time was from survey administration to

For peer review only

		the end of follow-up in December 2015 for more than 90% of the included individuals. (p 5-6)
Outcome data	15*	Report numbers of outcome events or summary measures over time<br>This is shown in Table 5 for both cause-specific and all-cause mortality outcomes. (p 26)
Main results	16	(*a*) Give unadjusted estimates and, if applicable, confounder-adjusted estimates and their precision (eg, 95% confidence interval). Make clear which confounders were adjusted for and why they were included<br>Estimates (hazard ratios) and their 95% confidence intervals are presented, using the structure of the table or a footnote to clarify the adjustments included. (p 24-26) Unadjusted estimates were deemed to be less informative than minimally adjusted estimates given the strong association of demographic variables such as age with cardiovascular mortality, though a tiered adjustment strategy is used to illustrate the robustness of our null results as we add socioeconomic and contextual covariates.
		(*b*) Report category boundaries when continuous variables were categorized<br>Continuous variables were either maintained in models as continuous or dichotomized as any versus none (=0 versus >0). (p 8-9)
		(*c*) If relevant, consider translating estimates of relative risk into absolute risk for a meaningful time period<br>This was deemed unnecessary to inform interpretation of our largely null results.
Other analyses	17	Report other analyses done—e.g., analyses of subgroups and interactions, and sensitivity analyses<br>Other analyses are discussed in the last two paragraphs of the Results section, and illustrated either within the main tables or in supplementary materials. (p 11-12)
**Discussion**		
Key results	18	Summarise key results with reference to study objectives<br>Key results are summarized in the first paragraph of the Discussion section. (p 12)
Limitations	19	Discuss limitations of the study, taking into account sources of potential bias or imprecision. Discuss both direction and magnitude of any potential bias<br>Limitations are discussed under the subheading "Strengths and limitations," with attention to whether sources of bias are likely to occur and whether the magnitude would likely overturn the observed patterns and conclusions reached. (p 15)
Interpretation	20	Give a cautious overall interpretation of results considering objectives, limitations, multiplicity of analyses, results from similar studies, and other relevant evidence<br>Caution and the context of prior work are used in discussing our findings and their possible implications. (p 16)
Generalisability	21	Discuss the generalisability (external validity) of the study results<br>The geographic context within the continental US is discussed a strength (with the study design and use of weighting designed to approximate associations that would be observed in a nationally representative sample of adults). (p 14) However, selection bias and measurement challenges related to this national scope are also discussed among limitations. (p 15)
**Other information**		
Funding	22	Give the source of funding and the role of the funders for the present study and, if applicable, for the original study on which the present article is based<br>A disclaimer and acknowledgements of state and federal funding are provided. (p 17)

*Give information separately for exposed and unexposed groups.

For peer review only - http://bmjopen.bmj.com/site/about/guidelines.xhtml
For peer review only

Note: An Explanation and Elaboration article discusses each checklist item and gives methodological background and published examples of transparent reporting. The STROBE checklist is best used in conjunction with this article (freely available on the Web sites of PLoS Medicine at http://www.plosmedicine.org/, Annals of Internal Medicine at http://www.annals.org/, and Epidemiology at http://www.epidem.com/). Information on the STROBE Initiative is available at http://www.strobe-statement.org.

For peer review only

For peer review only - http://bmjopen.bmj.com/site/about/guidelines.xhtml
